# Integrative Analysis of Hippocampus Gene Expression Profiles Identifies Network Alterations in Aging and Alzheimer’s Disease

**DOI:** 10.3389/fnagi.2018.00153

**Published:** 2018-05-23

**Authors:** Vinay Lanke, S. T. R. Moolamalla, Dipanjan Roy, P. K. Vinod

**Affiliations:** ^1^Center for Computational Natural Sciences and Bioinformatics, International Institute of Information Technology, Hyderabad, Hyderabad, India; ^2^Cognitive Brain Dynamics Lab, National Brain Research Centre, Gurgaon, India

**Keywords:** neurodegenerative disease, aging, hippocampus, glial cells, co-expression network, PPI network, graph theory

## Abstract

Alzheimer’s disease (AD) is a neurodegenerative disorder contributing to rapid decline in cognitive function and ultimately dementia. Most cases of AD occur in elderly and later years. There is a growing need for understanding the relationship between aging and AD to identify shared and unique hallmarks associated with the disease in a region and cell-type specific manner. Although genomic studies on AD have been performed extensively, the molecular mechanism of disease progression is still not clear. The major objective of our study is to obtain a higher-order network-level understanding of aging and AD, and their relationship using the hippocampal gene expression profiles of young (20–50 years), aging (70–99 years), and AD (70–99 years). The hippocampus is vulnerable to damage at early stages of AD and altered neurogenesis in the hippocampus is linked to the onset of AD. We combined the weighted gene co-expression network and weighted protein–protein interaction network-level approaches to study the transition from young to aging to AD. The network analysis revealed the organization of co-expression network into functional modules that are cell-type specific in aging and AD. We found that modules associated with astrocytes, endothelial cells and microglial cells are upregulated and significantly correlate with both aging and AD. The modules associated with neurons, mitochondria and endoplasmic reticulum are downregulated and significantly correlate with AD than aging. The oligodendrocytes module does not show significant correlation with neither aging nor disease. Further, we identified aging- and AD-specific interactions/subnetworks by integrating the gene expression with a human protein–protein interaction network. We found dysregulation of genes encoding protein kinases (FYN, SYK, SRC, PKC, MAPK1, ephrin receptors) and transcription factors (FOS, STAT3, CEBPB, MYC, NFKβ, and EGR1) in AD. Further, we found genes that encode proteins with neuroprotective function (14-3-3 proteins, PIN1, ATXN1, BDNF, VEGFA) to be part of the downregulated AD subnetwork. Our study highlights that simultaneously analyzing aging and AD will help to understand the pre-clinical and clinical phase of AD and aid in developing the treatment strategies.

## Introduction

Aging is associated with decline in cognitive abilities, including memory and executive function supported by prefrontal cortex and hippocampus (Yankner et al., 2008). The age-related cognitive decline is characterized by synaptic changes/loss of synapses in the absence of significant neuron loss and microglial dysfunction (Morrison and Hof, 1997; Von Bernhardi et al., 2015b). AD is a neurodegenerative disorder that in most cases occur in elderly and later years (Herrup, 2010). AD is characterized by the progressive loss of neurons contributing to the rapid decline in cognitive function and ultimately dementia. AD is linked to the accumulation of amyloid plaques and neurofibrillary tangles (NFTs), which are aggregates of amyloid β (Aβ) and hyperphosphorylated Tau protein, respectively (Ballatore et al., 2007; Selkoe and Hardy, 2016). Both aging and AD affect different regions of the brain and specific regions are more vulnerable than others (Braak and Braak, 1995; Morrison and Hof, 1997; Wang et al., 2016). Hippocampus is especially vulnerable to damage at early stages of AD (Mu and Gage, 2011). Further, emerging evidence suggests that altered neurogenesis in the adult hippocampus might play a role in the onset of AD (Ertaylan et al., 2014; Hollands et al., 2016). There is a growing need for understanding the relationship between aging and neurodegenerative disease to identify shared and unique hallmarks associated with the disease progression in a region and cell-type specific manner.

Genome-wide expression profiling of hippocampus have been widely used to investigate the aging and pathogenesis of AD in human post-mortem brain tissues (Colangelo et al., 2002; Blalock et al., 2004, 2011; Liang et al., 2007, 2008; Berchtold et al., 2008; Miller et al., 2013; Hokama et al., 2014; Wang et al., 2016). Berchtold et al. (2008) showed that gene expression changes in aging are sexually dimorphic with hippocampus showing minimal changes compared to superior-frontal gyrus, entorhinal cortex, and postcentral gyrus. Transcriptional studies on AD show dysfunction of synaptic signaling, energy metabolism, inflammation, protein misfolding, glutamate-mediated excitotoxicity, dysregulation of intracellular calcium, cell proliferation, myelin–axon interactions, cytoskeletal dynamics and lipid metabolism (Colangelo et al., 2002; Blalock et al., 2004; Lin and Beal, 2006; Miller et al., 2008; Bamburg and Bloom, 2009; Supnet and Bezprozvanny, 2010; Talantova et al., 2013). Further, network-level analysis have been used to identify local and global alterations from high-throughput gene expression datasets (Miller et al., 2008, 2013; Liang et al., 2012; Kikuchi et al., 2013; Talwar et al., 2014; Wang et al., 2016). Miller et al. (2008) identified two functional modules related to energy metabolism and synaptic plasticity that are conserved between aging and AD by comparing the samples obtained from frontal cortex and hippocampus. However, these studies focused on analyzing aging (young vs. aging) and AD (aging vs. AD) datasets individually.

On the other hand, few studies have compared the gene expression profiles in young, aging, and AD (Cribbs et al., 2012; Berchtold et al., 2013). The gene expression profiling of immune/inflammation-specific genes has shown that major changes occur in aging compared to AD with majority of genes significantly upregulated in hippocampus, superior-frontal gyrus and postcentral gyrus. A subset of genes changes progressively across aging and AD in hippocampus and superior-frontal gyrus. The synaptic genes were downregulated in aging with most genes showing progressive downregulation across aging and AD. Further, genes associated with neuronal loss, glial activation, and lipid metabolism are shown to increase with chronological age (Podtelezhnikov et al., 2011). However, in AD, these genes are reported to be prematurely expressed along with genes related to the protein folding and cell adhesion. A comparison of expression profiles of genes encoding respiratory oxidative phosphorylation (OXPHOS) complexes (I-V) in the hippocampus of young (20–59), aging (69–99), MCI and AD groups has shown that aging contributes to the decline of nuclear OXPHOS genes in AD (Mastroeni et al., 2017). These overlapping features between aging and AD suggest that a combined network analysis of both will help to understand the relationship between them and to generate insights on the mechanism(s) that promote disease progression.

The major objective of our study is to obtain a higher-order network-level understanding of aging and AD, and their relationship using the hippocampal gene expression profiles of young (20–50 years), aging (70–99 years), and AD (70–99 years). We combined the weighted gene co-expression network and weighted PPI network-level approaches to study the transition from young to aging to AD. The co-expression network analysis clusters genes into functional modules based on the gene expression profiles and helps to identify core biological processes and pathways associated with the sample group. The weighted PPI network uses the expression data to calculate edge weights in the network and helps to identify edges and subnetworks that are significantly affected between groups. We found modules associated with neuron, glial and endothelial cells in the co-expression network of young, aging, and AD. These modules significantly correlate with both aging and AD. We also show the preservation of these modules in five different hippocampus datasets of AD. Mapping the gene expression to PPI network helped to identify the upregulated and downregulated subnetworks of aging and AD.

## Materials and Methods

### Microarray Data Acquisition and Pre-processing

**FIGURE 1:**
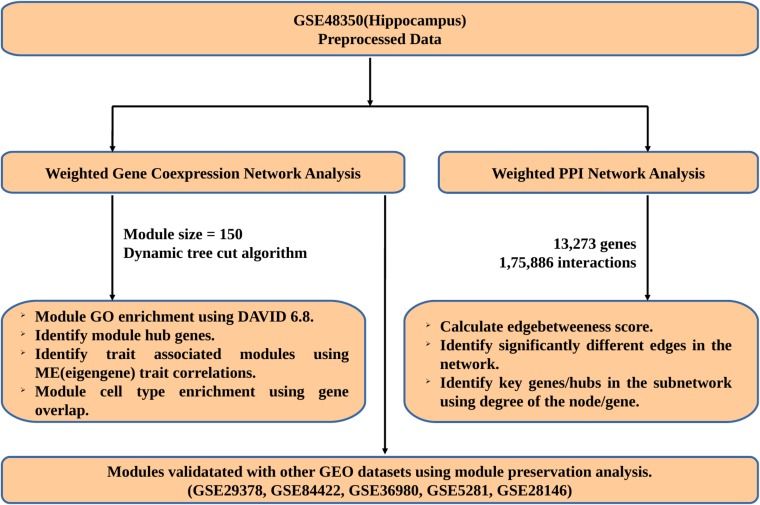
Workflow used to study young, aging, and AD.

Gene expression data of different age groups and AD samples with accession no: GSE48350 was downloaded from Gene Expression Omnibus (GEO)^1^. This dataset is obtained using Affymetrix Human Genome U133 Plus 2.0 Array and includes 62 samples obtained from the hippocampus. We processed the data using robust multichip average (RMA) algorithm, which performs background correction, quartile normalization and summarization of microarray dataset (Irizarry et al., 2003). The hippocampal data was divided into three groups depending on age and disease – Young (17 samples – 20–50 years), Aged (21 samples – 70–99 years), and AD (18 samples – 70–99 years). The probes were annotated using hgu133plus2.db package and probes with no annotation and multiple gene annotations were removed from the analysis. In case of multiple probes for the same gene, probe with high Interquartile Range (IQR) values was retained for further analysis. The workflow used in this study is shown in **Figure 1**.

### Weighted Gene Co-expression Network Analysis (WGCNA)

The WGCNA package in R was used to construct a signed co-expression network from the expression data (Langfelder and Horvath, 2008). WGCNA was performed using 18,754 varying genes (IQR > 0.2) across conditions to simplify computation and to eliminate non-varying genes (Zhang and Horvath, 2005; Oldham et al., 2006). Pearson correlations between all gene pairs were calculated to form the correlation matrix. To retain the sign of correlation, a linear transformation of correlation was performed using the Eq. (1).

$${\text{S}}_{\text{ij}}=\frac{1+\text{cor}|{\text{x}}_{\text{i}},\;{\text{x}}_{\text{j}}|}{2}$$

The correlation matrix was converted to adjacency matrix using the function, $a_{\text{ij}}=S_{\text{ij}}^{\beta }$ (Zhang and Horvath, 2005). A scale free topology criterion was used to choose power β. The square of the correlation (*R*^2^) between log(p(k)) and log(k) is used to measure how well a network satisfies a scale free topology (Horvath, 2011). p(k) is the frequency distribution of the connectivity k. The relationship between *R*^2^ and β is characterized by a saturation curve. The lowest power β = 18 (where the saturation is reached) was considered for the analysis (**Supplementary Figure S1**).

The resultant adjacency matrix was transformed into topological overlap matrix (TOM) and a dendrogram was constructed using 1-TOM as a distance measure (Zhang and Horvath, 2005). The genes were clustered into modules using a dynamic tree-cut algorithm with a minimum module size of 150 (Langfelder et al., 2008). Singular Value Decomposition (SVD) was used to obtain the ME, which represents the maximum amount of variation of module genes (Langfelder and Horvath, 2007). The ME expression value was correlated with age, group stages (Young – 0; Aging – 1; AD – 2), and AD (Young and Aging-0, AD-1) to identify modules associated with aging and disease. The hub genes were identified based on the intramodular connectivity (k_IM_) (Horvath, 2011). The GO terms and KEGG pathways associated with each module were obtained using DAVID version 6.8 (Dennis et al., 2003). Benjamini–Hochberg corrected *p*-value (adj *p*-value < 0.05) was used to find the significant GO terms and KEGG pathways.

In addition, cell-type specific gene lists obtained from Wang et al. (2016) was used to determine modules enriched for specific cell-type (astrocytes, endothelial, neurons, microglial, and oligodendrocytes). The overlap between module and cell-type gene lists was tested using Fisher’s exact test and a *p*-value cut off < 0.05 was used to identify cell-type specific modules. This was performed using the GeneOverlap package in R (Shen and Sinai, 2013). We also checked the overlap between modules and differential expressed genes (DEGs). We performed empirical Bayes statistical analysis using LIMMA R-package (Ritchie et al., 2015) to obtain DEGs between young vs. aging, aging vs. AD, and young vs. AD. The genes with fold change ≥1.5 and Benjamini–Hochberg corrected p-value <0.05 were considered as DEGs.

The reliability of the identified modules was checked by performing module preservation analysis using hippocampal test datasets of whole tissue: GSE1297, GSE36980, GSE84422, GSE29378 (both CA1 and CA3) and neuron enriched samples: GSE28146, GSE5281. These datasets were independently proces-sed depending on the platform (**Supplementary Table S1**) and module genes were used as an input to quantify the extent of preservation in each datasets. A Z_summary_ statistics proposed by Langfelder et al. (2011) was used to find the extent of preservation. The following thresholds for Z_summary_ were used: no preservation (Z_summary_ < 2), weak to moderate evidence of preservation (2 < Z_summary_< 10), and strong evidence of module preservation (Z_summary_ > 10) (Langfelder et al., 2011).

### Weighted PPI Network Analysis

A comprehensive human PPI network constructed by Sambarey et al. (2017) was used for the network analysis. This PPI network comprises of 17,062 proteins (nodes) and 168,237 directed interactions (edges) based on their functional annotations and 40,522 bidirectional interactions representing the formation of structural complexes (Sambarey et al., 2017). We overlapped our gene list with PPI network and removed the non-interacting edges. The resultant network consists of 13,273 nodes/genes and 175,886 edges/interactions. A weighted PPI network was constructed by mapping the gene expression to PPI network. The normalized signal intensity of a gene was used as condition-specific (young, aging, and disease) node weight (N_i_). The edge weight (W_ij_) between two nodes (N_i_ and N_j_) was calculated using the Eq. (2) (Sambarey et al., 2017).

$${\text{W}}_{\text{ij}}=\text{Inverse}\sqrt{{\text{N}}_{\text{i}}\times {\text{N}}_{\text{j}}}$$

### Graph Theory Approach

The edge betweenness centrality measure was computed using igraph R package (Girvan and Newman, 2002). It is defined as total number of shortest paths that go through an edge in the given network and highlights the importance of certain edges in establishing connection between many pairs of nodes. Each edge of the network is associated with edge betweenness score and can be compared across different networks. The edge betweenness scores were used to identify the differential connected edges between young vs. aging, aging vs. AD, and young vs. AD by performing paired t-tests and multiple testing correction with Benjamini-Hochberg method (Benjamini and Hochberg, 1995). An edge betweenness score difference of 2000 (adj *p*-value < 0.05) was considered as differentially connected.

## Results

### Co-expression Network Analysis of Progression Network: Young to Aging to AD

**FIGURE 2:**
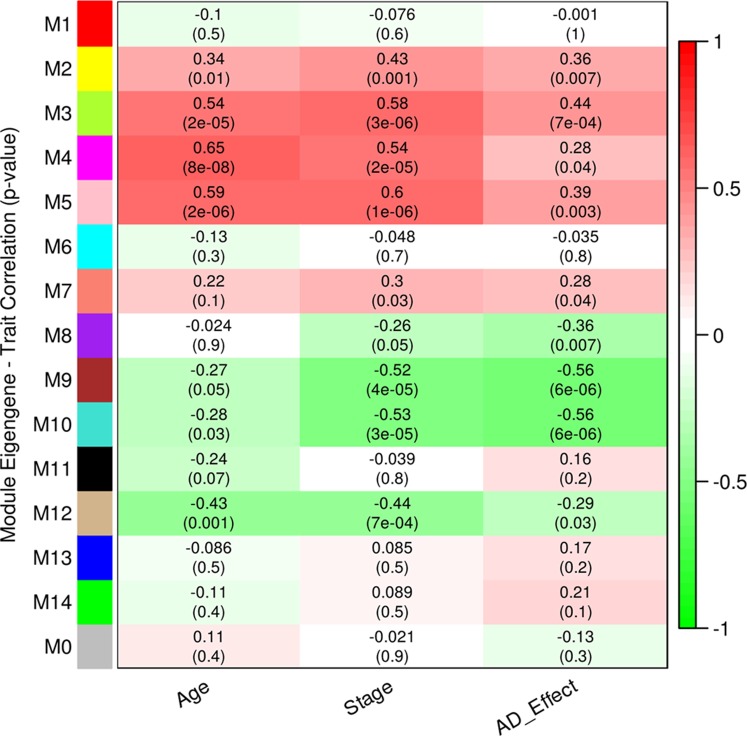
Correlation between module eigengene (ME) expression value and age, stage (0-young, 1-aging, 2-AD), AD (young and aging-0, AD-1) for each module. Pearson correlation is reported with the *p*-value given inside the bracket.

**FIGURE 3:**
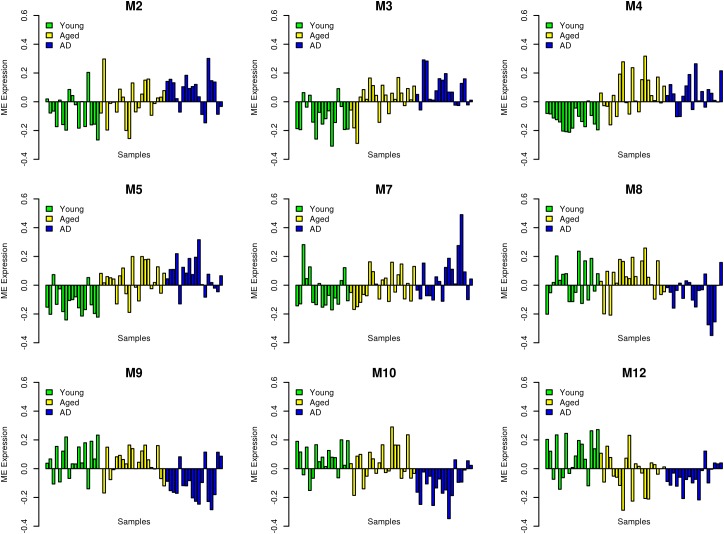
Module eigengene (ME) expression values (*y*-axis) across samples (*x*-axis). The samples are grouped into young (green), aging (yellow) and AD (blue).

**FIGURE 4:**
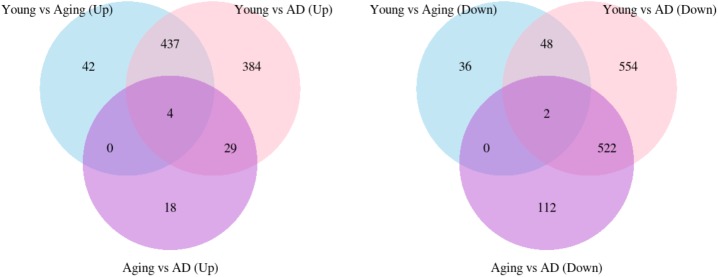
The overlap of upregulated and downregulated DEGs between young vs. aging, young vs. AD and aging vs. AD.

We performed WGCNA using 18,754 genes to identify and characterize modules that are related to aging and AD. A co-expression network was constructed independent of clinical information, age and gender using all the samples. We found 15 modules of co-expressed genes (**Supplementary Figure S2**). The ME expression values of M2 (yellow), M3 (green yellow), M4 (magenta), and M5 (pink) show positive correlation with both aging and AD (**Figure 2**). M4 and M5 modules have a strong correlation with respect to aging while M2 and M3 modules have a strong correlation with respect to AD. Further, the ME expression values of M9 (brown), M10 (turquoise), and M12 (tan) show negative correlation with both aging and AD. However, M9 and M10 modules have a strong correlation with respect to AD compared to aging. **Figure 3** shows the ME expression value for individual samples, which are grouped into young, aging, and AD. This grouping shows that there are inter-group differences in the ME expression value. The ME expression value indicates that genes of module M2, M3, M4, and M5 are upregulated while genes of modules M9, M10 and M12 are downregulated in the transition from young to aging to AD. We found DEGs and mapped it to the modules. There are 569, 687 and 1980 DEGs between young vs. aging, aging vs. AD, and young vs. AD, respectively. **Figure 4** shows the number of overlapping and age/disease-specific upregulated and downregulated DEGs in these paired comparisons. These DEGs are distributed among the modules that significantly correlate with aging and AD (**Supplementary Table S2**).

We also grouped samples into male and female, and explored the correlation of modules to aging and AD in a gender-specific manner. The ME expression values of M4, M5, and M7 show difference between young and aging depending on the gender (**Supplementary Figure S3**). We observed that the extent of correlation differs slightly due to the gender effect for the modules M4 (female correlation = 0.77, *p*-value = 1E-04; male correlation = 0.63, *p*-value = 0.004) and M5 (female correlation = 0.72, *p*-value = 5E-04; male correlation = 0.5, *p*-value = 0.03) while the module M7 correlated with aging in the female group only (correlation = 0.46, *p*-value = 0.05). The module M8 also shows differences between female and male of younger group (**Supplementary Figure S3**). These differences in the modules M7 and M8 make them to significantly correlate with AD only (**Figure 2**).

**FIGURE 5:**
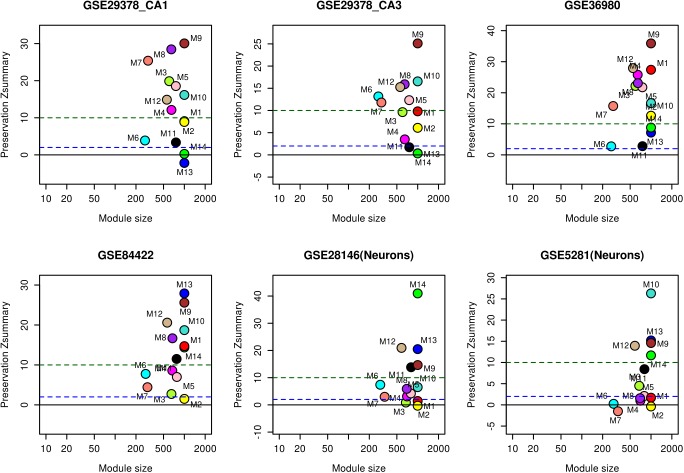
Module preservation analysis with hippocampal datasets.

We analyzed the overlap of cell-type specific genes with modules (**Table 1**). This analysis showed that modules M3 and M11 (black) are linked to astrocytes, modules M8, M9, and M12 are linked to neurons, the module M5 is linked mostly to endothelial cells, the module M4 is linked to microglia, and the module M1 (red) is related to oligodendrocytes. On the other hand, modules M10 and M2 show less significance with the cell-type. Further, the module-specific to oligodendrocytes does not show significant correlation with neither aging nor AD (**Figure 2**). We also performed module preservation analysis with independent datasets of hippocampus whole tissue: GSE1297, GSE36980, GSE84422, GSE29378 (both CA1 and CA3) and neuron enriched samples (GSE28146, GSE5281). We observed that most of the aging- and AD- related modules identified in our study show moderate to high preservation (**Figure 5**). The modules M1, M8, M9, M10 and M12 show a high preservation compared to other modules. The modules-specific to neurons (M8, M9 and M12), microglia (M4), endothelial cells (M5) and astrocytes (M3) are preserved in multiple datasets. Since both neuron and glial cells are affected together in AD, we suggest that neuron-glial interactions might be affected in AD. Further, the module M10, which shows less significance with the cell-type (**Table 1**), is also preserved in the neuron enriched datasets (**Figure 5**).

**Table 1:** The overlap between cell-type specific genes and modules.

Module	Astrocytes	Endothelial	Microglia	Neurons	Oligodendrocytes
M1	1.0	0.998	1.0	1.0	**1.56E-74**
M2	0.99	0.667	1.0	1.0	0.95
M3	**1.75E-81**	0.244	1.0	1.0	1.0
M4	0.998	0.489	**1.17E-109**	1.0	1.0
M5	**4.64E-03**	**5.60E-31**	0.489	1.0	0.989
M6	0.723	0.292	0.496	0.99	1.0
M7	0.949	1.0	1.0	0.897	1.0
M8	0.947	0.929	0.983	**1.73E-25**	0.98
M9	1.0	1.0	1.0	**3.42E-248**	1.0
M10	1.0	1.0	1.0	0.998	1.0
M11	**2.25E-06**	0.18	1.0	1.0	0.077
M12	0.995	1.0	1.0	**1.39E-09**	1.0
M13	1.0	1.0	1.0	1.0	1.0
M14	1.0	0.96	1.0	1.0	1.0

*p-values are obtained using Fisher’s exact test. The significant values are shows in bold.*

We characterized the biological processes and KEGG pathways associated with modules using DAVID for the functional enrichment analysis. We found that microglia module M4 is associated with the biological process inflammatory response and KEGG pathways phagosome, Toll-like receptor signaling and cytokine-cytokine receptor interactions. Further, it is associated with the cellular component MHC class II protein complex (**Table 2**). The hub genes of the module M4 include: TYROBP, TREM2, ITGB2, MYO1F, C1QA, C1QB, C1QC, and TGFB1. The endothelial cell module M5 is also associated with the inflammatory response and KEGG pathways TNF signaling, complement and coagulation cascades, and HIF-1 signaling pathway. It is associated with the cellular component MHC class I protein complex and extracellular matrix (ECM) protein complex. The hub genes of the module M5 include: TNFRSF1A, MSN, CLIC1, and IFITM2.

**Table 2:** Enrichment of Gene Ontology (GO) terms and KEGG pathways associated with aging and AD-specific modules.

Module (genes)	KEGG pathway	Biological process	Cellular components	Hub genes
M2 (2072)	Ribosome (1.7E-10), Spliceosome (1.2E-2), RNA transport (2.4E-4^∗^)	rRNA processing (2.4E-13), mRNA splicing (3.6E-5), mRNA processing (1.1E-4)	Nucleolus (1.6E-10), Ribosome (4.1E-6)	TFEB, PAN2, ARHGAP17

M3 (675)	Fatty acid degradation (8.2E-3), Hippo signaling (2.7E-2), PPAR signaling (9.7E-4^∗^)	Cell adhesion (4.1E-2), Oxidation–reduction process (3.0E-4^∗^), Fatty acid beta-oxidation (8.5E-4^∗^)	Extracellular exosome (1.5E-2), Focal adhesion (3.9E-4^∗^), Extracellular space (8.9E-4^∗^)	CDC42EP4, EZR, ARHGEF26, TCF7L1, SOX9, ARHGEF6

M4 (701)	Phagosome (4.3E-8), Toll-like receptor signaling (3.1E-6), Cytokine–cytokine receptor interaction (2.7E-4)	Inflammatory response (1.4E-18), Signal transduction (4.0E-12), Toll-like receptor signaling (7.6E-8)	MHC class II protein complex (1.8E-6), Integral component of membrane (2.6E-3), Phagocytic vesicle membrane (4.3E-3)	TYROBP, TREM2, ITGB2, MYO1F, C1Qs, TGFB1

M5 (798)	TNF signaling (1.9E-6), Complement and coagulation cascades (5.6E-5), HIF-1 signaling (4.1E-4)	Inflammatory response (4.8E-16), Response to LPS (1.5E-8), Cellular response to TNF (2.2E-8)	Extracellular matrix (3.5E-7), Extracellular exosome (5.8E-7), MHC class 1 complex (1.4E-4)	TNFRSF1A, MSN, CLIC1, IFITM2

M7 (333)	ECM-receptor interaction (6.4E-3^∗^), Focal adhesion (3.6E-2^∗^)	Outer dynein arm assembly (6.4E-12), Inner dynein arm assembly (3.8E-9), Cilium morphogenesis (9.0E-9)	Axoneme (1.3E-18), Motile cilium (5.5E-17)	ZMYND10, ARMC3, CFAP43

M8 (695)	Axon guidance (5.9E-3), Oxytocin signaling (1.6E-3^∗^), Rap1 signaling (4.8E-3^∗^)	Calcium ion transport (8.1E-3^∗^), Potassium ion transport (9.9E-3^∗^), Dendritic spine morphogenesis (1.1E-2^∗^)	Cell junction (9.7E-9), Postsynaptic density (6.4E-8), Dendritic spine (9.1E-5)	ICAM5, PRKCG, JPH3, SPTBN2

M9 (2377)	Synaptic vesicle (8.3E-10), Glutamatergic synapse (3.8E-5), Long term potentiation (4.2E-3)	Chemical synaptic transmission (2.3E-16), Neurotransmitter secretion (1.5E-6), Nervous system development (5.8E-5)	Neuron projection (2.5E-10), Dendrite (1.9E-10), Axon (4.9E-9)	UCHL1, STMN2, SYN1, SYT5, SNAP91, PAK3

M10 (2660)	Oxidative phosphorylation (1.8E-19), Proteasome (6.6E-11), Spliceosome (7.5E-7), Protein processing in ER (3.8E-5)	Mitochondria electron transport (1.4E-12), Protein folding (9.3E-9)	Mitochondrion (2.4E-62), Mitochondrial matrix (2.4E-19), Proteasome complex (3.4E-9), Ribosome (2.5E-8)	NDUFAB1, VDAC3, ATP5G3, COPS4, RTCA, POP4

M12 (584)	Retrograde endocannabinoid signaling (4.8E-4), Circadian entrainment (7.1E-3), Glutamatergic synapse (7.2E-3), GABAergic synapse (2.9E-2)	Peptidyl-serine phosphorylation (2.9E-4^∗^), Neuron cell–cell adhesion (2.1E-3^∗^)	Postsynaptic density (3.5E-2), Postsynaptic membrane (2.9E-2)	YWHAZ, GADP1, SYNJ1, MAPK9, G3BP2, ATP6AP2

*Benjamini-Hochberg corrected p-values are given inside the bracket. ^∗^Represents uncorrected p-value.*

The astrocyte module M3 is associated with the biological process cell adhesion and KEGG pathways fatty acid degradation and HIPPO signaling pathway (**Table 2**). The genes related to actin cytoskeleton (EZR, CDC42EP4, ARHGEF26, ARHGEF6) are the hub genes. Further, we found two transcriptional factors TCF7L1 and SOX9 as hubs genes. SOX9 is highly expressed in astrocytes and plays a role in glial fate specification (Sun et al., 2017). The upregulated module M2 is associated with the biological process RNA splicing and KEGG pathways ribosome, spliceosome, and RNA transport. A key hub gene of the module M2 is TFEB, PAN2, and ARHGAP17.

The neuron module M9 is associated with biological processes chemical synaptic transmission, neurotransmitter secretion and nervous system development, and KEGG pathways synaptic vesicle, chemical synapses (glutamatergic, cholinergic, GABAergic, serotonergic, and dopaminergic) and long-term potentiation (**Table 2**). The key cellular components include neuron projection, dendrite (dendrite morphogenesis), axon (axongenesis/axon guidance) and post synaptic density. The hub genes of the neuron module M9 include SYN1, STMN2, SYT5, SNAP91, PAK3, UCHL1, and UBE2K. Interestingly, these hub genes are significantly downregulated in AD compared to aging. The downregulation of glutamatergic synapse together with inhibitory GABAergic synapse suggests that there is an alteration in the excitation and inhibition (E/I) balance in the progression of AD. The neuron module M12 is also related to chemical synapses and post synaptic density. The genes within this module show gradual downregulation with aging and AD. We found that the neuron module M12 hub genes GADP1, YWHAZ, SYNJ1, and MAPK9, decrease significantly with aging while the hub genes G3BP2 and ATP6AP2 decrease significantly with AD. The neuron module M8 also includes more genes involved in the axon guidance and post synaptic density. The overlapping biological processes between modules M8, M9, and M12 suggests that different patterns of downregulation of genes within same biological processes.

Further, the module M10 is another downregulated module related to AD and it is associated with mitochondria, ribosome, and protein folding (**Table 2**). The KEGG pathways include oxidative phosphorylation, proteasome, spliceosome, aminoacyl tRNA biosynthesis and protein processing in endoplasmic reticulum (ER) involving protein targeting, ER-associated degradation and ubiquitin ligase complex. This suggests that mitochondria and ER functions are affected in AD. The hub genes include NDUFAB1, VDAC3, ATP5G3, COPS4, RTCA, and POP4. This downregulated module is also associated with the RNA transport, translation, and splicing. Similarly, the module M2 is also associated with ribosome and RNA splicing, but it shows positive correlation with aging and AD (**Figure 2** and **Table 2**) suggesting a complex pattern of gene expression related to these cellular processes.

In the oligodendrocyte module M1, we note that most genes are downregulated with aging but in AD the extent of downregulation decrease and in some patients this module is upregulated (**Supplementary Figure S4**). In this module, the myelin associated proteins (MBP, MOB, and MOG) are downregulated in aging together with negative regulators (LINGO1) of myelination and oligodendrocytes precursor differentiation. This suggests a dynamic homeostasis of myelin damage and repair, which can mask its consequences in aging and pathogenesis of AD.

### Mapping Gene Expression to Human Protein–Protein Interaction Network: Graph Theoretical Study

We integrated the gene expression of young, aging, and AD with the PPI network to obtain weighted PPI networks. The integrated PPI networks were used to identify active/inactive interactions between young vs. aging and young vs. AD using the edge betweenness network measure. Only those interactions with an edge betweenness value difference of 2000 and adj *p*-value < 0.05 were considered to be altered interactions. These interactions formed the upregulated and downregulated subnetworks of aging and AD (**Supplementary Figures S5**, **S6**). In AD, the number of nodes and interactions increased (**Supplementary Figure S7** and **Data Sheet S1**). However, we observed that most of these alterations in AD increase the degree of existing nodes of aging subnetwork, which lead to an increase in the number of hubs. This suggests that common process/nodes are dysregulated to different extent in aging and AD. Based on the node degree, we identified hub genes and their interactions in aging and AD PPI subnetworks.

**FIGURE 6:**
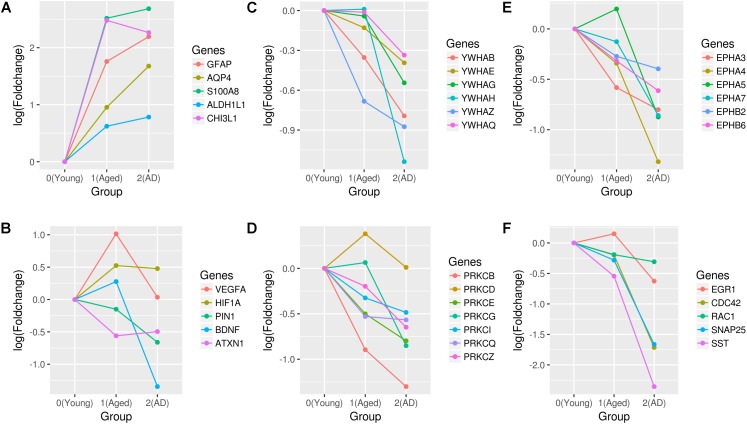
Expression profiles of genes associated with aging and AD. The fold changes of **(A)** astrocytes markers, **(B)** neuroprotective genes (VEGFA, HIF1A, PIN1, BDNF, and ATXN1), **(C)** 14-3-3 proteins, **(D)** PRKC isoforms, **(E)** ephrin receptors, and **(F)** synapses and actin cytoskeleton genes (EGR1, CDC42, RAC1, SNAP25, and SST) are shown. Fold changes are calculated with respect to the expression of young group.

We found nodes CD44, VEGFA, HIF1A, VIM, FOS, CEBPB, CDKN1A, SHC1, TGFβ1, and SYK as hub genes of the upregulated aging subnetwork. CD44 is expressed in both glial and neuronal cells and it is associated with astrocytes migration and differentiation, astrogliosis, oligodendrocytes differentiation, inflammatory response, dendritic arborization, actin polymerization, and synaptic transmission (Dzwonek and Wilczynski, 2015). Similarly, another identified gene Vimentin (VIM) is also highly expressed in astrocytes (Ferrer, 2017). Consistently, we also observe an increase in the expression of astrocyte markers, GFAP, S100A8, ALDH1L1, and CHI3L1 with aging (**Figure 6A**). Specifically, CHI3L is identified as a biomarker for reactive astrocytes linked to the inflammation.

VEGFA interaction with HIF1A is upregulated in aging. We also found key interactions between HIF1A, PFKFB3, and LDHA, which regulate the metabolic switch toward aerobic glycolysis. This is also further supported by the upregulation of hexokinase 2 (HK2) and pyruvate dehydrogenase kinase 1 (PDK1) genes with aging compared to the young. DNA damage responsive gene CDKN1A is upregulated in aging along with its interacting partner GADD45B. Further, the upregulated aging subnetwork also includes hub genes related to immune and inflammatory response (CEBPB, FOS, STAT3, TGFβ1, and SYK). In young vs. AD, we found that the upregulated subnetwork expands with newer interactions and hub genes also include RXRA, ACTB, RELA, NFKBIA, FYN, MYC, and YBX1. NFKβ subunit, RELA interactions increase compared to the aging subnetwork and is related to the immune response. We found interactions involving YBX1, MYC, MAX, MXI1, SGK1, and FOXO3 in AD that are linked to cell proliferation and cell death decision-making. The repressor element 1-silencing transcription factor (REST) interactions are also part of the upregulated subnetwork. REST is linked to the stress resistance in aging and AD (Lu et al., 2014). Further, in the AD subnetwork, VEGFA is not a hub gene since the number of interactions decrease significantly compared to the aging subnetwork. We observed that the expression of VEGFA increase in aging but decrease in AD (**Figure 6B**), which suggests the possibility of vascular dysfunction in the hippocampus of AD.

The hub genes of downregulated aging subnetwork include MAPK1, RAD21, YWHAZ, ATXN1, SRC, CTCF, CALM1, and PRKCZ. In AD, the node degree of MAPK1, CALM1, YWHAZ, PRKCZ, SRC, and CTCF increases while that of RAD21 decreases. Further, we also found hub genes PIN1, YWHAH, YWHAG, YWHAQ, EGR1, CDC42, DNM1, SST, and SNAP25 that are specific for AD. YWHAZ, YWHAH, YWHAG, and YWHAQ encode proteins of the 14-3-3 family that are highly expressed in the brain tissue and are involved in the brain development, memory and learning (Qiao et al., 2014). We observed that genes of 14-3-3 family are downregulated in AD compared to aging (**Figure 6C**). Similarly, PIN1 is downregulated in AD, which is required for healthy aging and its deficiency leads to an early aging phenotype (Liou et al., 2003). In AD, we also observed that members of PKC family of serine/threonine kinase are downregulated together with genes encoding kinases, sarcoma tyrosine kinase (SRC) and ephrin receptors (**Figures 6D,E**). Recent evidence shows that these protein kinase activities decrease with the stages of AD (Rosenberger et al., 2016).

CDC42 interaction with GRB2 is downregulated, which has a preventive role in the cytoskeleton disassembly. Further, SNAP25 encoding a SNARE complex protein and DNM1 encoding a dynamin subfamily of GTP-binding protein, are downregulated affecting the synaptic vesicle exocytosis and endocytosis, respectively. The neuropeptide somatostatin (SST) gene is also downregulated, which suggest that the SST group of GABAergic interneurons are affected (**Figure 6F**). Furthermore, an interaction involving BDNF and a GABA related gene GAD2 is downregulated in the AD subnetwork.

## Discussion

In this study, we extend the work of Cribbs et al. (2012) and Berchtold et al. (2013) by performing large-scale network analyses of aging and AD using the gene expression profiles obtained from the post-mortem hippocampus samples. Earlier studies have shown that genes related to immune, energy metabolism and synapses are altered in aging and AD. Here, the network analysis revealed the organization of co-expression network into functional modules that are cell-type specific in aging and AD. We showed that the module associated with neurons, glial cells and endothelial cells are affected by both aging and AD (**Figure 2** and **Table 1**). The modules associated with neurons, mitochondria and ER are downregulated while modules associated with glial cells (microglial and astrocytes) and endothelial cells are upregulated (**Figure 3**). Both microglial and endothelial cell modules are upregulated to different extent in male and female (**Supplementary Figure S3**). Further, two modules (one of them is the neuron module M8) showed gender differences suggesting sexual dimorphism in the hippocampus as reported previously (Berchtold et al., 2008; Guebel and Torres, 2016). Further, some of the hub genes of these modules are implicated in aging and AD (**Table 3**).

**Table 3:** Module hub genes in aging and AD.

Gene	Role/function	Reference
TFEB	Involved in Aβ-induced pathogenesis of AD by regulating the autophagy-lysosome pathway	Zhang and Zhao, 2015
EZR	Role in immune synapse along with MSN	Ivetic and Ridley, 2004
TCF7L1	Mediates Wnt signaling pathway; Altered in AD patients in the hippocampus	Riise et al., 2015
SOX9	Glial fate specification	Sun et al., 2017
TYROBP	Activates immune response; genetic variants are risk factor for AD	Wang et al., 2016
TREM2	Activates immune response; genetic variants are risk factor for AD	Guerreiro et al., 2013
C1Qs	Associated with early synaptic loss in AD mice models	Stephan et al., 2013
TGFB1	Major role in the activation of microglia	Von Bernhardi et al., 2015a
ITGB2	Identified as one of key inflammatory gene in AD mice models	Wes et al., 2014
MSN	Role in immune synapse along with EZR; identified as highly expressed in the AD brain using proteomic analysis	Ivetic and Ridley, 2004; Begcevic et al., 2013
CLIC1	Identified as highly expressed in the AD brain using proteomic analysis; involved in Aβ induced generation of ROS	Milton et al., 2008; Begcevic et al., 2013
IFITM2	Identified as part of microglial sensome in aging with neuroprotective role	Hickman et al., 2013
TNFRSF1A	Identified as AD associated gene using genome wide haplotype association study	Shang et al., 2015
UCHL1	Regulates the production of Aβ by interacting with APP	Zhang et al., 2014
SNAP91	Role in vesicle mediated transport; downregulated in AD patients and AD mice models	Cao et al., 2010
PAK3	Reduced activity in AD patients and AD mice models	Zhao et al., 2006
NDUFAB1	Role in energy metabolism; downregulated in AD	Liang et al., 2008
YWHAZ	Identified as AD biomarker using proteomic analysis; reported as hub gene in aging and AD	Miller et al., 2008; Ho Kim et al., 2015
SYNJ1	Accelerate Aβ clearance and attenuates cognitive deterioration	Zhu et al., 2013
ATP6AP2	Downregulation induces neurodegeneration	Dubos et al., 2015

The upregulation of glial cells-associated modules in aging is consistent with the study by Cribbs et al. (2012) and Soreq et al. (2017). We found that the microglial module genes are significantly upregulated in young vs. aging than aging vs. AD (**Figures 2**, **3**). This suggests that the neuroinflammation is significantly associated with aging and precedes the development of AD. The upregulation of astrocyte module genes in aging and AD can reflect the neuroprotective role to relieve the stress on the aging neurons or deleterious processes in AD. This finding is consistent with a previous study that shows an increase in the expression of astrocyte genes in human aging and aging mouse models in the hippocampus (Hayakawa et al., 2007).

An increase in the expression of the endothelial cell module genes and VEGFA are also observed in aging. On the other hand, the expression of VEGFA decreased in AD (**Figure 6B**). Emerging evidences show vascular dysfunction in the development and progression of AD (De Strooper and Karran, 2016). The brain tissue of AD patients shows elevated apoptotic vascular cells. It is also shown that expressing VEGF in neurons restored the impaired memory in AD mice suggesting that VEGF is required to protect the integrity of the vasculature in aging (Religa et al., 2013). We also found evidence for aerobic glycolysis (HIF1A and its interactions) in aging using the PPI network analysis (**Supplementary Figure S5**). High brain lactate is shown to be the hallmark of aging, however, its role in maintaining the neuronal function during aging is not clear (Ross et al., 2010). Interestingly, studies have shown that Aβ resistance is mediated by aerobic glycolysis (Soucek et al., 2003; Newington et al., 2011).

Further, two neuron modules M8 and M9 are significantly downregulated with AD than aging and another neurons module M12 is downregulated as continuum of aging (**Figures 2**, **3**). We observed that both glutamatergic synapse and inhibitory GABAergic synapse are downregulated in AD. Previous studies have shown hyperexcitability in the early stage of AD (MCI), which can be due to the effect of losing the inhibitory synapse. This might lead to hyper- to hypo- excitability with the progression of AD (Berchtold et al., 2014; Saura et al., 2015). These results suggest that neurons-specific changes characterize disease state while glial cells-specific changes characterize aging. This is consistent with the observation that glial cells-specific genes predict the age than neurons-specific genes (Soreq et al., 2017). The complex interaction between glial cells in aging might form the early cellular phase of AD followed by the alteration in the E/I balance at the level of neurons.

We found genes that encode protein kinases as hubs of upregulated (FYN and SYK) and downregulated (SRC, MAPK1, PRKCZ) PPI subnetworks. FYN is a tyrosine kinase that is implicated in AD and its known targets are PTK2B and Tau (Kaufman et al., 2015). Its inhibition is shown to rescue the memory loss in the AD mice. SYK overexpression leads to Aβ accumulation and Tau hyperphosphorylation in AD (Paris et al., 2014). PRKCZ function is linked to memory consolidation and maintenance (Bonini et al., 2007). MAPK1 is involved in long-term potentiation and memory consolidation in the hippocampus. Increased levels of Aβ suppress the expression of MAPK1 (Liu et al., 2013). We also found the transcriptional dysregulation in both aging and AD. FOS (a subunit of transcriptional factor AP-1), STAT3 and CEBPB, which are involved in the immune response, are hub genes of the upregulated aging subnetwork. In the AD subnetwork, the degree of these nodes increases and more hub genes emerge, which include transcriptional activator MYC and regulators of NFKβ (RELA, NFKBIA). An increase in the expression of MYC is associated with neuronal cell death (Lee et al., 2011). EGR1 is part of downregulated AD subnetwork and it encodes a member of the immediate early gene (IEG) family of transcription factors involved in the regulation of synaptic plasticity (Duclot and Kabbaj, 2017).

Further, we found more proteins (14-3-3 proteins, PIN1, ATXN1, and BDNF) with neuroprotective function in aging to be part of the downregulated AD subnetwork (**Figure 6**). PIN1-dependent protein isomerization protects against NFTs and Aβ accumulation (Liou et al., 2003; Balastik et al., 2007). BDNF levels decrease in the serum and brain of AD, and it has a protective role against Aβ- and Tau-related neurodegeneration (Jiao et al., 2016). The loss of ATXN1 potentiates β-secretase cleavage of APP, which leads to an increase in Aβ levels (Zhang et al., 2010). This subnetwork also includes hub genes (CDC42) that are involved in the regulation of actin cytoskeleton. The downregulation of Rho family GTPases (CDC42 and RAC1) genes leads to synaptic loss in AD (Konietzny et al., 2017). We found both axon guidance and dendrite morphogenesis, which are dependent on the dynamics of cytoskeleton, to be affected (the neuron module M9). These processes are implicated in AD and evidences suggest that Tau might disrupt the dynamics of cytoskeleton leading to the synaptic loss in AD (Bamburg and Bloom, 2009).

In summary, our study provides network-level insights into the complex relationship between aging and AD. The co-expression network of young, aging, and AD helped to identify modules, pathways and genes that are stage-specific, cell-type specific and continuum in the hippocampus, which were unclear in the previous studies that focused on either aging or AD. We identified the genes and their interactions that protect aging brain from AD and that make it susceptible to AD. We also demonstrated the validity of our study by identifying pathways and genes that are previously implicated in aging and AD. Our study highlights that simultaneously analyzing aging and AD will help to understand the pre-clinical and clinical phase of AD and aid in developing treatment strategies. This study can be further extended to characterize the global and local alterations in the other areas of the brain in young, aging, and AD.

## Author Contributions

PKV and VL designed the study. VL and SM carried out the analysis. VL, SM, DR, and PKV analyzed the data, wrote the manuscript, and gave the final approval for publication.

## Conflict of Interest Statement

The authors declare that the research was conducted in the absence of any commercial or financial relationships that could be construed as a potential conflict of interest.

## Footnotes

1 https://www.ncbi.nlm.nih.gov/geo/

## Supplementary Material

DATA SHEET 1 Up-regulated and down-regulated interactions of young vs. aging, aging vs. AD and young vs. AD.

FIGURE S1 Scale free topology fit showing the relation between *R*^2^ and power (β) for the co-expression network. The curve saturates at β = 18.

FIGURE S2 Modular organization of the co-expression network. The modules are shown in different colors below the dendrogram. The gray module consists of genes not assigned to any module.

FIGURE S3 Module eigengene (ME) expression values (*y*-axis) across samples (*x*-axis). The female and male samples are separately grouped into young (green), aging (yellow) and AD (blue). Female group is shown on the left and male group is shown on the right.

FIGURE S4 Oligodendrocyte module eigengene (ME) expression values (*y*-axis) across samples (*x*-axis). The samples are grouped into young (green), aging (yellow) and AD (blue).

FIGURE S5 The upregulated **(A)** aging and **(B)** AD subnetworks. The significant interactions of young vs. aging, and young vs. AD obtained using edge betweenness network measure are shown. Genes/nodes with significant interactions are shown in green color.

FIGURE S6 The downregulated **(A)** aging and **(B)** AD subnetworks. The significant interactions of young vs. aging, and young vs. AD obtained using edge betweenness network measure are shown. Genes/nodes with significant interactions are shown in green color.

FIGURE S7 The overlap of upregulated and downregulated nodes and interactions between aging and AD subnetworks.

TABLE S1 Datasets used for the module preservation analysis.

TABLE S2 The distribution of DEGs in the different modules of co-expression network.

## Abbreviations

AD: Alzheimer’s disease
DAVID: Database for Annotation, Visualization and Integrated Discovery
DEG: differentially expressed gene
GO: Gene Ontology
LIMMA: Linear Models for Microarray
MCI: mild cognitive impairment
ME: module eigengene
PPI: protein–protein interaction
WGCNA: weighted gene co-expression network analysis

## References

[B1] Balastik M. Lim J. Pastorino L. Lu K. P. (2007). Pin1 in Alzheimer’s disease: multiple substrates, one regulatory mechanism? 1772 422–429. 10.1016/j.bbadis.2007.01.006 17317113 PMC1868500

[B2] Ballatore C. Lee V. M. Trojanowski J. Q. (2007). Tau-mediated neurodegeneration in Alzheimer’s disease and related disorders. 8 663–672. 10.1038/nrn2194 17684513

[B3] Bamburg J. R. Bloom G. S. (2009). Cytoskeletal pathologies of Alzheimer disease. 66 635–649. 10.1002/cm.20388 19479823 PMC2754410

[B4] Begcevic I. Kosanam H. Martinez-Morillo E. Dimitromanolakis A. Diamandis P. Kuzmanov U. (2013). Semiquantitative proteomic analysis of human hippocampal tissues from Alzheimer’s disease and age-matched control brains. 10:5. 10.1186/1559-0275-10-5 23635041 PMC3648498

[B5] Benjamini Y. Hochberg Y. (1995). Controlling the false discovery rate: a practical and powerful approach to multiple testing. 57 289–300.

[B6] Berchtold N. C. Coleman P. D. Cribbs D. H. Rogers J. Gillen D. L. Cotman C. W. (2013). Synaptic genes are extensively downregulated across multiple brain regions in normal human aging and Alzheimer’s disease. 34 1653–1661. 10.1016/j.neurobiolaging.2012.11.024 23273601 PMC4022280

[B7] Berchtold N. C. Cribbs D. H. Coleman P. D. Rogers J. Head E. Kim R. (2008). Gene expression changes in the course of normal brain aging are sexually dimorphic. 105 15605–15610. 10.1073/pnas.0806883105 18832152 PMC2563070

[B8] Berchtold N. C. Sabbagh M. N. Beach T. G. Kim R. C. Cribbs D. H. Cotman C. W. (2014). Brain gene expression patterns differentiate mild cognitive impairment from normal aged and Alzheimer’s disease. 35 1961–1972. 10.1016/j.neurobiolaging.2014.03.031 24786631 PMC4067010

[B9] Blalock E. M. Buechel H. M. Popovic J. Geddes J. W. Landfield P. W. (2011). Microarray analyses of laser-captured hippocampus reveal distinct gray and white matter signatures associated with incipient Alzheimer’s disease. 42 118–126. 10.1016/j.jchemneu.2011.06.007 21756998 PMC3163806

[B10] Blalock E. M. Geddes J. W. Chen K. C. Porter N. M. Markesbery W. R. Landfield P. W. (2004). Incipient Alzheimer’s disease: microarray correlation analyses reveal major transcriptional and tumor suppressor responses. 101 2173–2178. 10.1073/pnas.0308512100 14769913 PMC357071

[B11] Bonini J. S. Da Silva W. C. Bevilaqua L. R. Medina J. H. Izquierdo I. Cammarota M. (2007). On the participation of hippocampal PKC in acquisition, consolidation and reconsolidation of spatial memory. 147 37–45. 10.1016/j.neuroscience.2007.04.013 17499932

[B12] Braak H. Braak E. (1995). Staging of Alzheimer’s disease-related neurofibrillary changes. 16 271–278. 10.1016/0197-4580(95)00021-6 7566337

[B13] Cao Y. Xiao Y. Ravid R. Guan Z. Z. (2010). Changed clathrin regulatory proteins in the brains of Alzheimer’s disease patients and animal models. 22 329–342. 10.3233/JAD-2010-100162 20847448

[B14] Colangelo V. Schurr J. Ball M. J. Pelaez R. P. Bazan N. G. Lukiw W. J. (2002). Gene expression profiling of 12633 genes in Alzheimer hippocampal CA1: transcription and neurotrophic factor down-regulation and up-regulation of apoptotic and pro-inflammatory signaling. 70 462–473. 10.1002/jnr.10351 12391607

[B15] Cribbs D. H. Berchtold N. C. Perreau V. Coleman P. D. Rogers J. Tenner A. J. (2012). Extensive innate immune gene activation accompanies brain aging, increasing vulnerability to cognitive decline and neurodegeneration: a microarray study. 9:179. 10.1186/1742-2094-9-179 22824372 PMC3419089

[B16] Dennis G. Jr. Sherman B. T. Hosack D. A. Yang J. Gao W. (2003). DAVID: database for annotation, visualization, and integrated discovery. 4:P3 10.1186/gb-2003-4-5-p3 PMC193660 12734009

[B17] De Strooper B. Karran E. (2016). The cellular phase of Alzheimer’s disease. 164 603–615. 10.1016/j.cell.2015.12.056 26871627

[B18] Dubos A. Castells-Nobau A. Meziane H. Oortveld M. A. Houbaert X. Iacono G. (2015). Conditional depletion of intellectual disability and Parkinsonism candidate gene ATP6AP2 in fly and mouse induces cognitive impairment and neurodegeneration. 24 6736–6755. 10.1093/hmg/ddv380 26376863 PMC4634377

[B19] Duclot F. Kabbaj M. (2017). The role of early growth response 1 (EGR1) in brain plasticity and neuropsychiatric disorders. 11:35. 10.3389/fnbeh.2017.00035 28321184 PMC5337695

[B20] Dzwonek J. Wilczynski G. M. (2015). CD44: molecular interactions, signaling and functions in the nervous system. 9:175 10.3389/fncel.2015.00175 PMC4423434 25999819

[B21] Ertaylan G. Okawa S. Schwamborn J. C. Del Sol A. (2014). Gene regulatory network analysis reveals differences in site-specific cell fate determination in mammalian brain. 8:437. 10.3389/fncel.2014.00437 25565969 PMC4270183

[B22] Ferrer I. (2017). Diversity of astroglial responses across human neurodegenerative disorders and brain aging. 27 645–674. 10.1111/bpa.12538 28804999 PMC8029391

[B23] Girvan M. Newman M. E. (2002). Community structure in social and biological networks. 99 7821–7826. 10.1073/pnas.122653799 12060727 PMC122977

[B24] Guebel D. V. Torres N. V. (2016). Sexual dimorphism and aging in the human hyppocampus: identification, validation, and impact of differentially expressed genes by factorial microarray and network analysis. 8:229. 10.3389/fnagi.2016.00229 27761111 PMC5050216

[B25] Guerreiro R. Wojtas A. Bras J. Carrasquillo M. Rogaeva E. Majounie E. (2013). TREM2 variants in Alzheimer’s disease. 368 117–127. 10.1056/NEJMoa1211851 23150934 PMC3631573

[B26] Hayakawa N. Kato H. Araki T. (2007). Age-related changes of astorocytes, oligodendrocytes and microglia in the mouse hippocampal CA1 sector. 128 311–316. 10.1016/j.mad.2007.01.005 17350671

[B27] Herrup K. (2010). Reimagining Alzheimer’s disease–an age-based hypothesis. 30 16755–16762. 10.1523/JNEUROSCI.4521-10.2010 21159946 PMC3004746

[B28] Hickman S. E. Kingery N. D. Ohsumi T. K. Borowsky M. L. Wang L. C. Means T. K. (2013). The microglial sensome revealed by direct RNA sequencing. 16 1896–1905. 10.1038/nn.3554 24162652 PMC3840123

[B29] Hokama M. Oka S. Leon J. Ninomiya T. Honda H. Sasaki K. (2014). Altered expression of diabetes-related genes in Alzheimer’s disease brains: the Hisayama study. 24 2476–2488. 10.1093/cercor/bht101 23595620 PMC4128707

[B30] Ho Kim J. Franck J. Kang T. Heinsen H. Ravid R. Ferrer I. (2015). Proteome-wide characterization of signalling interactions in the hippocampal CA4/DG subfield of patients with Alzheimer’s disease. 5:11138. 10.1038/srep11138 26059363 PMC4462342

[B31] Hollands C. Bartolotti N. Lazarov O. (2016). Alzheimer’s disease and hippocampal adult neurogenesis: exploring shared mechanisms. 10:178 10.3389/fnins.2016.00178 PMC4853383 27199641

[B32] Horvath S. (2011). *Weighted Network Analysis: Applications in Genomics and Systems Biology*. New York, NY: Springer-Verlag. 10.1007/978-1-4419-8819-5

[B33] Irizarry R. A. Hobbs B. Collin F. Beazer-Barclay Y. D. Antonellis K. J. Scherf U. (2003). Exploration, normalization, and summaries of high density oligonucleotide array probe level data. 4 249–264. 10.1093/biostatistics/4.2.249 12925520

[B34] Ivetic A. Ridley A. J. (2004). Ezrin/radixin/moesin proteins and Rho GTPase signalling in leucocytes. 112 165–176. 10.1111/j.1365-2567.2004.01882.x 15147559 PMC1782489

[B35] Jiao S. S. Shen L. L. Zhu C. Bu X. L. Liu Y. H. Liu C. H. (2016). Brain-derived neurotrophic factor protects against tau-related neurodegeneration of Alzheimer’s disease. 6:e907. 10.1038/tp.2016.186 27701410 PMC5315549

[B36] Kaufman A. C. Salazar S. V. Haas L. T. Yang J. Kostylev M. A. Jeng A. T. (2015). Fyn inhibition rescues established memory and synapse loss in Alzheimer mice. 77 953–971. 10.1002/ana.24394 25707991 PMC4447598

[B37] Kikuchi M. Ogishima S. Miyamoto T. Miyashita A. Kuwano R. Nakaya J. (2013). Identification of unstable network modules reveals disease modules associated with the progression of Alzheimer’s disease. 8:e76162. 10.1371/journal.pone.0076162 24348898 PMC3858171

[B38] Konietzny A. Bar J. Mikhaylova M. (2017). Dendritic actin cytoskeleton: structure, functions, and regulations. 11:147. 10.3389/fncel.2017.00147 28572759 PMC5435805

[B39] Langfelder P. Horvath S. (2007). Eigengene networks for studying the relationships between co-expression modules. 1:54. 10.1186/1752-0509-1-54 18031580 PMC2267703

[B40] Langfelder P. Horvath S. (2008). WGCNA: an R package for weighted correlation network analysis. 9:559. 10.1186/1471-2105-9-559 19114008 PMC2631488

[B41] Langfelder P. Luo R. Oldham M. C. Horvath S. (2011). Is my network module preserved and reproducible? 7:e1001057. 10.1371/journal.pcbi.1001057 21283776 PMC3024255

[B42] Langfelder P. Zhang B. Horvath S. (2008). Defining clusters from a hierarchical cluster tree: the dynamic tree cut package for R. 24 719–720. 10.1093/bioinformatics/btm563 18024473

[B43] Lee H. P. Kudo W. Zhu X. Smith M. A. Lee H. G. (2011). Early induction of c-Myc is associated with neuronal cell death. 505 124–127. 10.1016/j.neulet.2011.10.004 22005580 PMC3234683

[B44] Liang D. Han G. Feng X. Sun J. Duan Y. Lei H. (2012). Concerted perturbation observed in a hub network in Alzheimer’s disease. 7:e40498. 10.1371/journal.pone.0040498 22815752 PMC3398025

[B45] Liang W. S. Dunckley T. Beach T. G. Grover A. Mastroeni D. Walker D. G. (2007). Gene expression profiles in anatomically and functionally distinct regions of the normal aged human brain. 28 311–322. 10.1152/physiolgenomics.00208.2006 17077275 PMC2259385

[B46] Liang W. S. Reiman E. M. Valla J. Dunckley T. Beach T. G. Grover A. (2008). Alzheimer’s disease is associated with reduced expression of energy metabolism genes in posterior cingulate neurons. 105 4441–4446. 10.1073/pnas.0709259105 18332434 PMC2393743

[B47] Lin M. T. Beal M. F. (2006). Mitochondrial dysfunction and oxidative stress in neurodegenerative diseases. 443 787–795. 10.1038/nature05292 17051205

[B48] Liou Y. C. Sun A. Ryo A. Zhou X. Z. Yu Z. X. Huang H. K. (2003). Role of the prolyl isomerase Pin1 in protecting against age-dependent neurodegeneration. 424 556–561. 10.1038/nature01832 12891359

[B49] Liu T. Ren D. Zhu X. Yin Z. Jin G. Zhao Z. (2013). Transcriptional signaling pathways inversely regulated in Alzheimer’s disease and glioblastoma multiform. 3:3467. 10.1038/srep03467 24322672 PMC4894382

[B50] Lu T. Aron L. Zullo J. Pan Y. Kim H. Chen Y. (2014). REST and stress resistance in ageing and Alzheimer’s disease. 507 448–454. 10.1038/nature13163 24670762 PMC4110979

[B51] Mastroeni D. Khdour O. M. Delvaux E. Nolz J. Olsen G. Berchtold N. (2017). Nuclear but not mitochondrial-encoded oxidative phosphorylation genes are altered in aging, mild cognitive impairment, and Alzheimer’s disease. 13 510–519. 10.1016/j.jalz.2016.09.003 27793643 PMC5967608

[B52] Miller J. A. Oldham M. C. Geschwind D. H. (2008). A systems level analysis of transcriptional changes in Alzheimer’s disease and normal aging. 28 1410–1420. 10.1523/JNEUROSCI.4098-07.2008 PMC2902235 18256261

[B53] Miller J. A. Woltjer R. L. Goodenbour J. M. Horvath S. Geschwind D. H. (2013). Genes and pathways underlying regional and cell type changes in Alzheimer’s disease. 5:48. 10.1186/gm452 23705665 PMC3706780

[B54] Milton R. H. Abeti R. Averaimo S. Debiasi S. Vitellaro L. Jiang L. (2008). CLIC1 function is required for beta-amyloid-induced generation of reactive oxygen species by microglia. 28 11488–11499. 10.1523/JNEUROSCI.2431-08.2008 18987185 PMC6671295

[B55] Morrison J. H. Hof P. R. (1997). Life and death of neurons in the aging brain. 278 412–419. 10.1126/science.278.5337.412 9334292

[B56] Mu Y. Gage F. H. (2011). Adult hippocampal neurogenesis and its role in Alzheimer’s disease. 6:85. 10.1186/1750-1326-6-85 22192775 PMC3261815

[B57] Newington J. T. Pitts A. Chien A. Arseneault R. Schubert D. Cumming R. C. (2011). Amyloid beta resistance in nerve cell lines is mediated by the Warburg effect. 6:e19191. 10.1371/journal.pone.0019191 21541279 PMC3082554

[B58] Oldham M. C. Horvath S. Geschwind D. H. (2006). Conservation and evolution of gene coexpression networks in human and chimpanzee brains. 103 17973–17978. 10.1073/pnas.0605938103 17101986 PMC1693857

[B59] Paris D. Ait-Ghezala G. Bachmeier C. Laco G. Beaulieu-Abdelahad D. Lin Y. (2014). The spleen tyrosine kinase (Syk) regulates Alzheimer amyloid-beta production and Tau hyperphosphorylation. 289 33927–33944. 10.1074/jbc.M114.608091 25331948 PMC4256331

[B60] Podtelezhnikov A. A. Tanis K. Q. Nebozhyn M. Ray W. J. Stone D. J. Loboda A. P. (2011). Molecular insights into the pathogenesis of Alzheimer’s disease and its relationship to normal aging. 6:e29610. 10.1371/journal.pone.0029610 22216330 PMC3247273

[B61] Qiao H. Foote M. Graham K. Wu Y. Zhou Y. (2014). 14-3-3 proteins are required for hippocampal long-term potentiation and associative learning and memory. 34 4801–4808. 10.1523/JNEUROSCI.4393-13.2014 24695700 PMC3972712

[B62] Religa P. Cao R. Religa D. Xue Y. Bogdanovic N. Westaway D. (2013). VEGF significantly restores impaired memory behavior in Alzheimer’s mice by improvement of vascular survival. 3:2053. 10.1038/srep02053 23792494 PMC3690383

[B63] Riise J. Plath N. Pakkenberg B. Parachikova A. (2015). Aberrant Wnt signaling pathway in medial temporal lobe structures of Alzheimer’s disease. 122 1303–1318. 10.1007/s00702-015-1375-7 25680440

[B64] Ritchie M. E. Phipson B. Wu D. Hu Y. Law C. W. Shi W. (2015). limma powers differential expression analyses for RNA-sequencing and microarray studies. 43 e47. 10.1093/nar/gkv007 25605792 PMC4402510

[B65] Rosenberger A. F. Hilhorst R. Coart E. Garcia Barrado L. Naji F. Rozemuller A. J. (2016). Protein kinase activity decreases with higher Braak stages of Alzheimer’s disease pathology. 49 927–943. 10.3233/JAD-150429 26519433 PMC4927853

[B66] Ross J. M. Oberg J. Brene S. Coppotelli G. Terzioglu M. Pernold K. (2010). High brain lactate is a hallmark of aging and caused by a shift in the lactate dehydrogenase A/B ratio. 107 20087–20092. 10.1073/pnas.1008189107 21041631 PMC2993405

[B67] Sambarey A. Devaprasad A. Baloni P. Mishra M. Mohan A. Tyagi P. (2017). Meta-analysis of host response networks identifies a common core in tuberculosis. 3:4. 10.1038/s41540-017-0005-4 28649431 PMC5445610

[B68] Saura C. A. Parra-Damas A. Enriquez-Barreto L. (2015). Gene expression parallels synaptic excitability and plasticity changes in Alzheimer’s disease. 9:318. 10.3389/fncel.2015.00318 26379494 PMC4548151

[B69] Selkoe D. J. Hardy J. (2016). The amyloid hypothesis of Alzheimer’s disease at 25 years. 8 595–608. 10.15252/emmm.201606210 27025652 PMC4888851

[B70] Shang Z. Lv H. Zhang M. Duan L. Wang S. Li J. (2015). Genome-wide haplotype association study identify TNFRSF1A, CASP7, LRP1B, CDH1 and TG genes associated with Alzheimer’s disease in Caribbean Hispanic individuals. 6 42504–42514. 10.18632/oncotarget.6391 26621834 PMC4767448

[B71] Shen L. Sinai M. (2013). *GeneOverlap: Test and Visualize Gene Overlaps. R Package Version 1.14.0*. Available at: http://shenlab-sinai.github.io/shenlab-sinai/

[B72] Soreq L. Rose J. Soreq E. Hardy J. Trabzuni D. Cookson M. R. (2017). Major shifts in glial regional identity are a transcriptional hallmark of human brain aging. 18 557–570. 10.1016/j.celrep.2016.12.011 28076797 PMC5263238

[B73] Soucek T. Cumming R. Dargusch R. Maher P. Schubert D. (2003). The regulation of glucose metabolism by HIF-1 mediates a neuroprotective response to amyloid beta peptide. 39 43–56. 10.1016/S0896-6273(03)00367-2 12848931

[B74] Stephan A. H. Madison D. V. Mateos J. M. Fraser D. A. Lovelett E. A. Coutellier L. (2013). A dramatic increase of C1q protein in the CNS during normal aging. 33 13460–13474. 10.1523/JNEUROSCI.1333-13.2013 23946404 PMC3742932

[B75] Sun W. Cornwell A. Li J. Peng S. Osorio M. J. Aalling N. (2017). SOX9 is an astrocyte-specific nuclear marker in the adult brain outside the neurogenic regions. 37 4493–4507. 10.1523/JNEUROSCI.3199-16.2017 28336567 PMC5413187

[B76] Supnet C. Bezprozvanny I. (2010). The dysregulation of intracellular calcium in Alzheimer disease. 47 183–189. 10.1016/j.ceca.2009.12.014 20080301 PMC2834825

[B77] Talantova M. Sanz-Blasco S. Zhang X. Xia P. Akhtar M. W. Okamoto S. (2013). Abeta induces astrocytic glutamate release, extrasynaptic NMDA receptor activation, and synaptic loss. 110 E2518–E2527. 10.1073/pnas.1306832110 23776240 PMC3704025

[B78] Talwar P. Silla Y. Grover S. Gupta M. Agarwal R. Kushwaha S. (2014). Genomic convergence and network analysis approach to identify candidate genes in Alzheimer’s disease. 15:199. 10.1186/1471-2164-15-199 24628925 PMC4028079

[B79] Von Bernhardi R. Cornejo F. Parada G. E. Eugenin J. (2015a). Role of TGFbeta signaling in the pathogenesis of Alzheimer’s disease. 9:426 10.3389/fncel.2015.00426 PMC4623426 26578886

[B80] Von Bernhardi R. Eugenin-Von Bernhardi L. Eugenin J. (2015b). Microglial cell dysregulation in brain aging and neurodegeneration. 7:124. 10.3389/fnagi.2015.00124 26257642 PMC4507468

[B81] Wang M. Roussos P. Mckenzie A. Zhou X. Kajiwara Y. Brennand K. J. (2016). Integrative network analysis of nineteen brain regions identifies molecular signatures and networks underlying selective regional vulnerability to Alzheimer’s disease. 8:104. 10.1186/s13073-016-0355-3 27799057 PMC5088659

[B82] Wes P. D. Easton A. Corradi J. Barten D. M. Devidze N. Decarr L. B. (2014). Tau overexpression impacts a neuroinflammation gene expression network perturbed in Alzheimer’s disease. 9:e106050. 10.1371/journal.pone.0106050 25153994 PMC4143352

[B83] Yankner B. A. Lu T. Loerch P. (2008). The aging brain. 3 41–66. 10.1146/annurev.pathmechdis.2.010506.092044 18039130

[B84] Zhang B. Horvath S. (2005). A general framework for weighted gene co-expression network analysis. 4:Article17. 10.2202/1544-6115.1128 16646834

[B85] Zhang C. Browne A. Child D. Divito J. R. Stevenson J. A. Tanzi R. E. (2010). Loss of function of ATXN1 increases amyloid beta-protein levels by potentiating beta-secretase processing of beta-amyloid precursor protein. 285 8515–8526. 10.1074/jbc.M109.079079 20097758 PMC2838273

[B86] Zhang M. Cai F. Zhang S. Song W. (2014). Overexpression of ubiquitin carboxyl-terminal hydrolase L1 (UCHL1) delays Alzheimer’s progression *in vivo*. 4:7298. 10.1038/srep07298 25466238 PMC4252905

[B87] Zhang Y. D. Zhao J. J. (2015). TFEB participates in the Abeta-induced pathogenesis of Alzheimer’s disease by regulating the autophagy-lysosome pathway. 34 661–668. 10.1089/dna.2014.2738 26368054

[B88] Zhao L. Ma Q. L. Calon F. Harris-White M. E. Yang F. Lim G. P. (2006). Role of p21-activated kinase pathway defects in the cognitive deficits of Alzheimer disease. 9 234–242. 10.1038/nn1630 16415866

[B89] Zhu L. Zhong M. Zhao J. Rhee H. Caesar I. Knight E. M. (2013). Reduction of synaptojanin 1 accelerates Abeta clearance and attenuates cognitive deterioration in an Alzheimer mouse model. 288 32050–32063. 10.1074/jbc.M113.504365 24052255 PMC3814799

