# Supplementary figures and images for: Integrative Analysis of Hippocampus Gene Expression Profiles Identifies Network Alterations in Aging and Alzheimer’s Disease
Source: Front Aging Neurosci. 2018 May 23;10:153. doi: 10.3389/fnagi.2018.00153 (PMC5974201; doi:10.3389/fnagi.2018.00153)

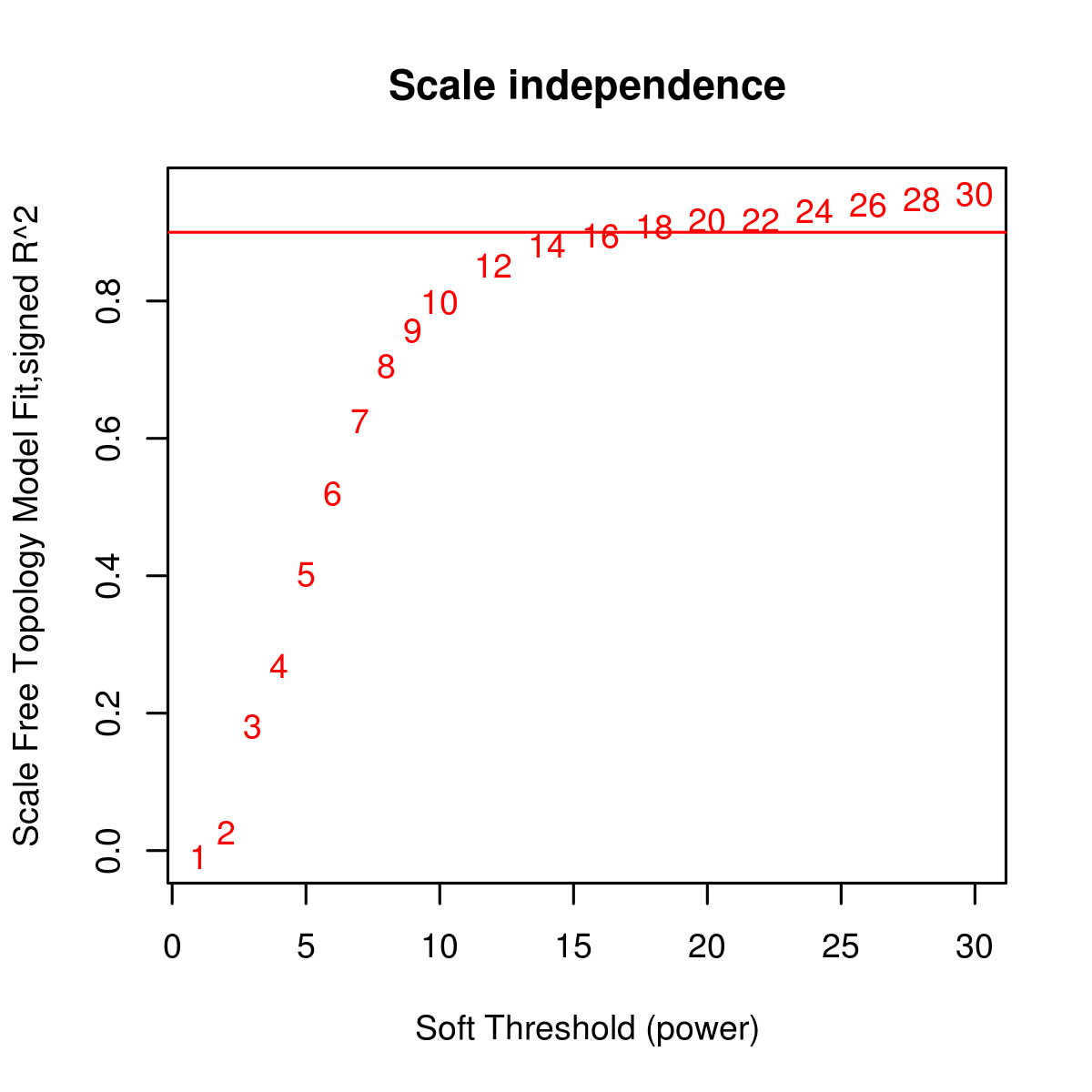

Supplement: FIGURE S1 — Scale free topology fit showing the relation between R2 and power (β) for the co-expression network. The curve saturates at β = 18. [file Image_1.TIFF]

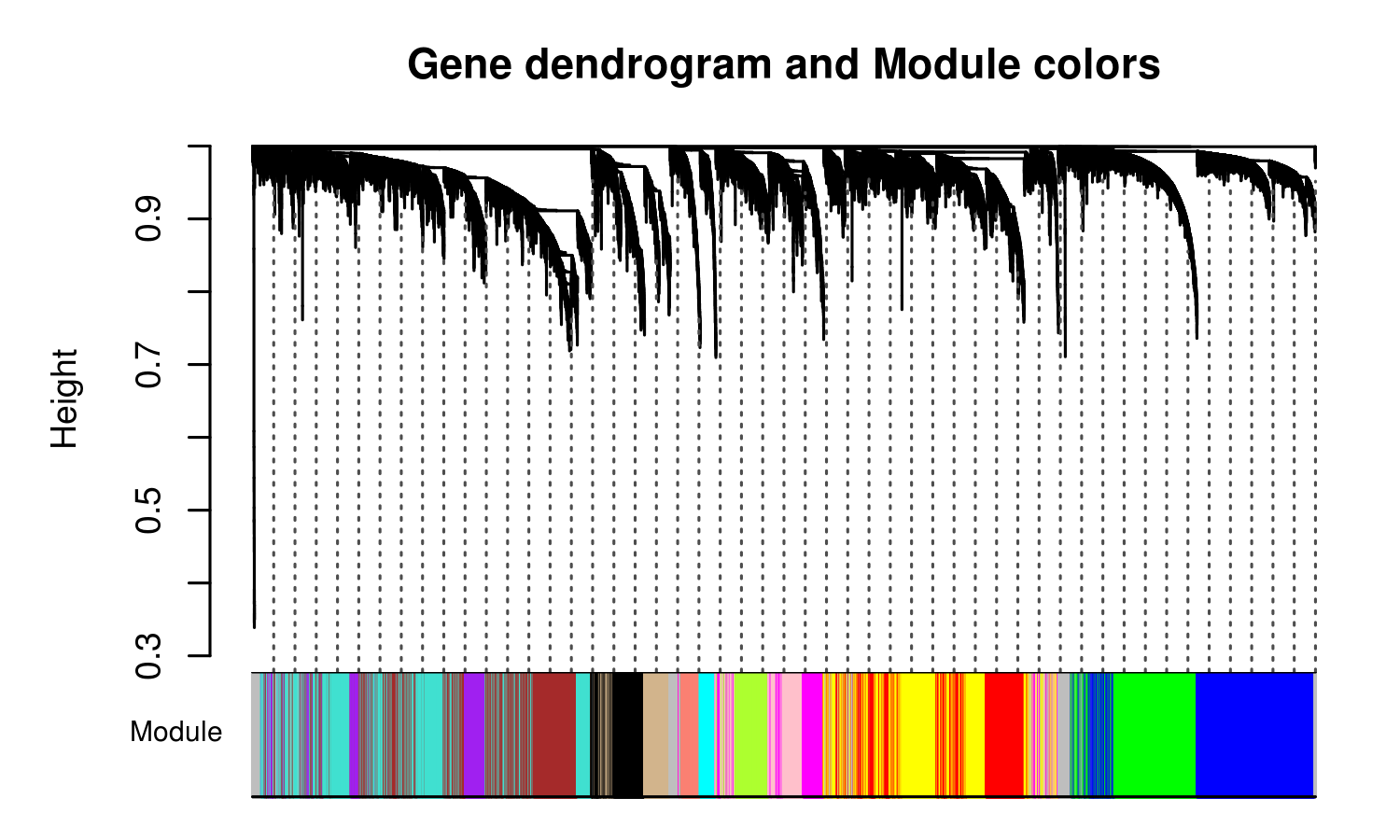

Supplement: FIGURE S2 — Modular organization of the co-expression network. The modules are shown in different colors below the dendrogram. The gray module consists of genes not assigned to any module. [file Image_2.TIFF]

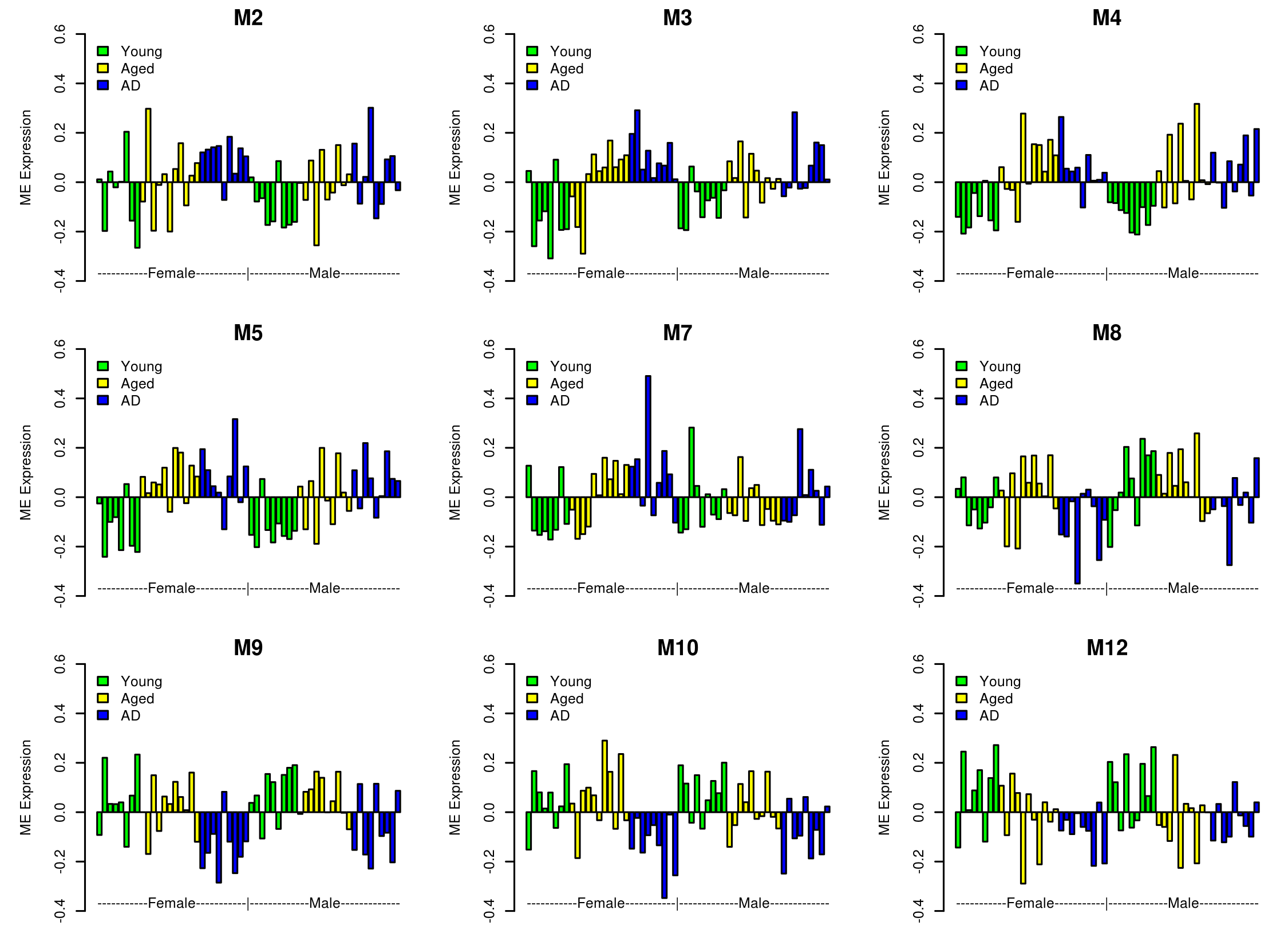

Supplement: FIGURE S3 — Module eigengene (ME) expression values (y-axis) across samples (x-axis). The female and male samples are separately grouped into young (green), aging (yellow) and AD (blue). Female group is shown on the left and male group is shown on the right. [file Image_3.TIFF]

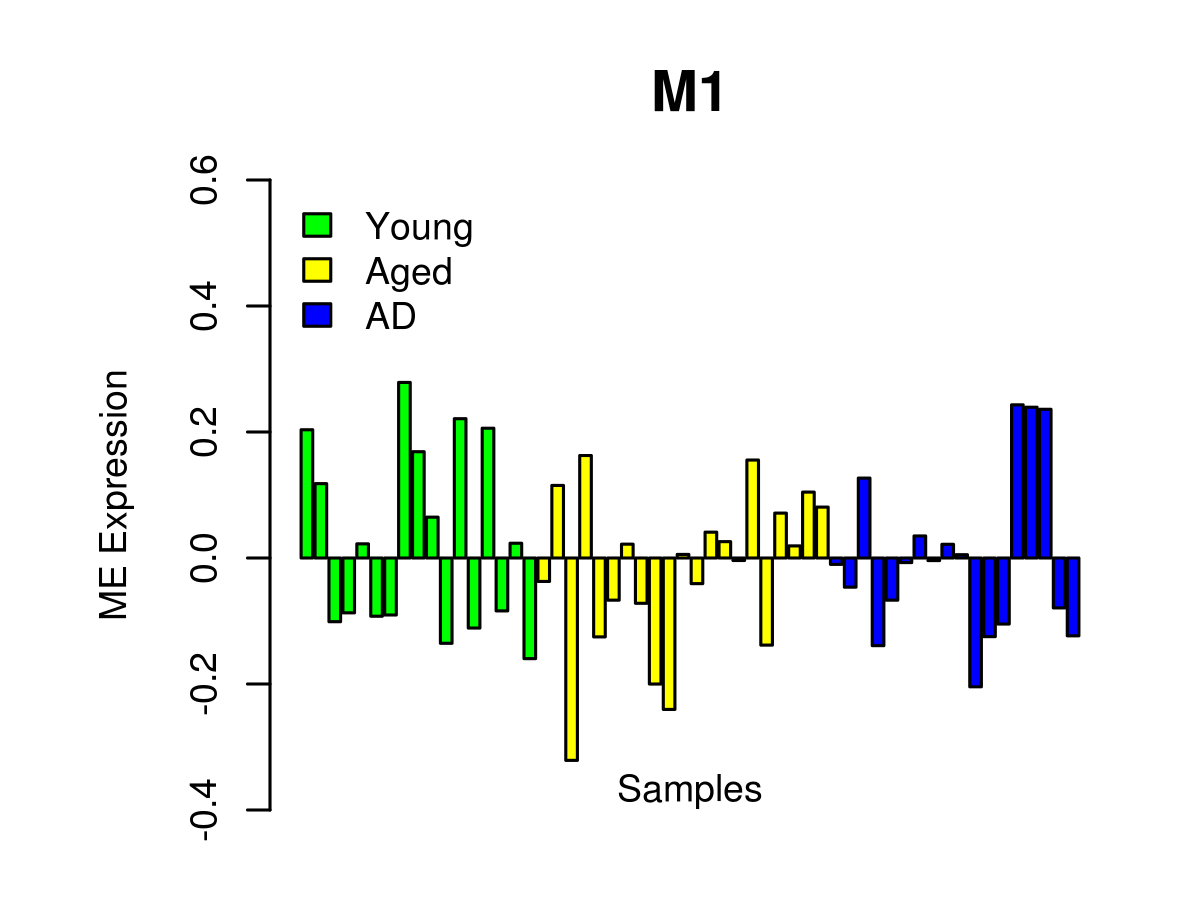

Supplement: FIGURE S4 — Oligodendrocyte module eigengene (ME) expression values (y-axis) across samples (x-axis). The samples are grouped into young (green), aging (yellow) and AD (blue). [file Image_4.TIFF]

A

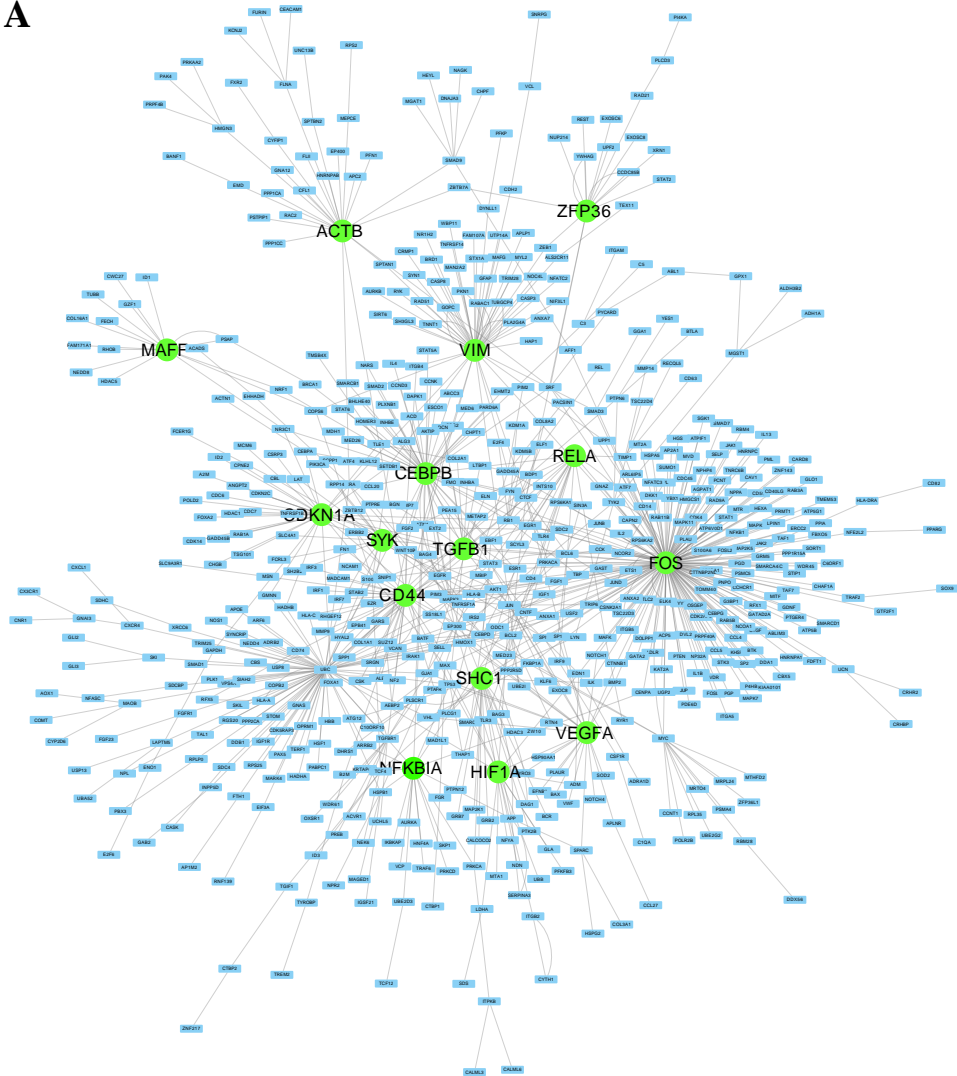

B

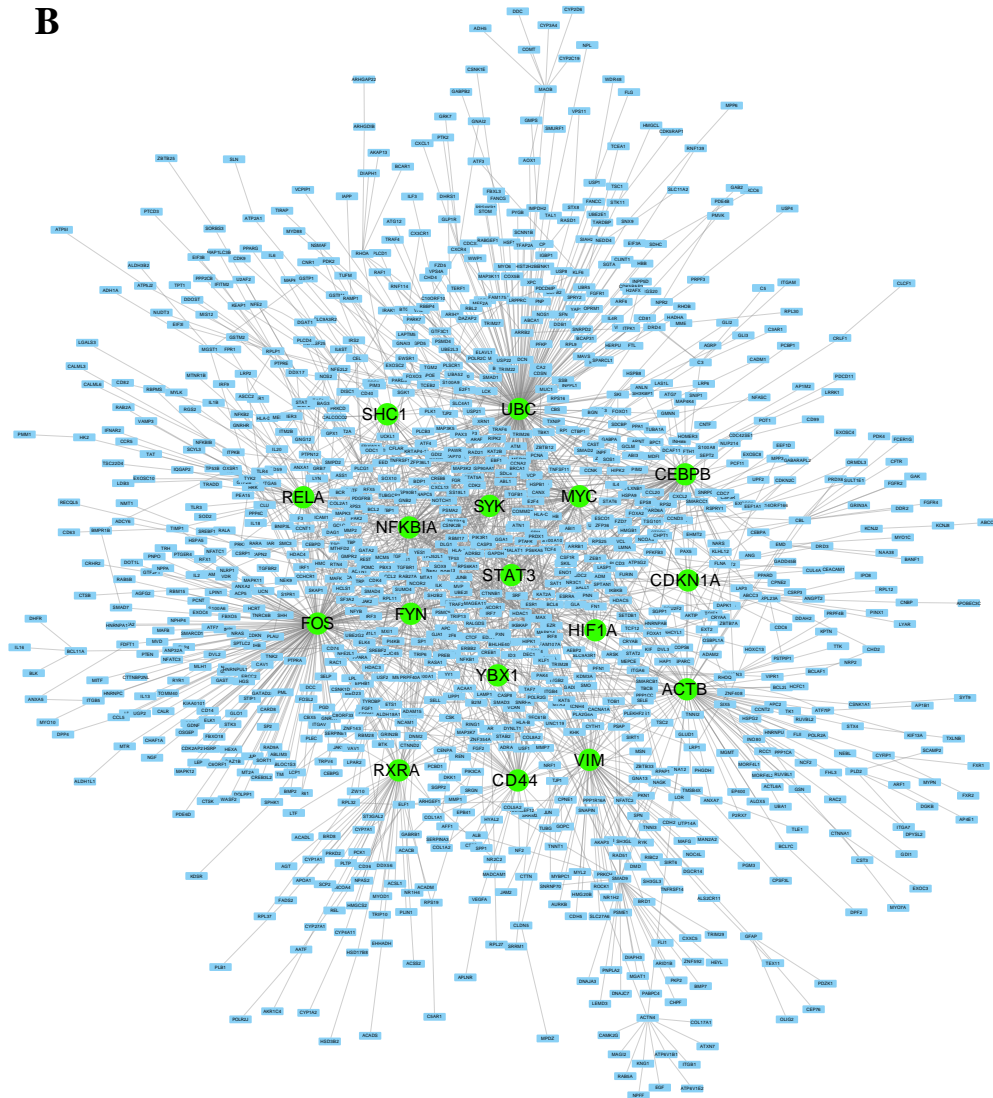

Supplement: FIGURE S5 — The upregulated (A) aging and (B) AD subnetworks. The significant interactions of young vs. aging, and young vs. AD obtained using edge betweenness network measure are shown. Genes/nodes with significant interactions are shown in green color. [file Image_5.PDF]

A

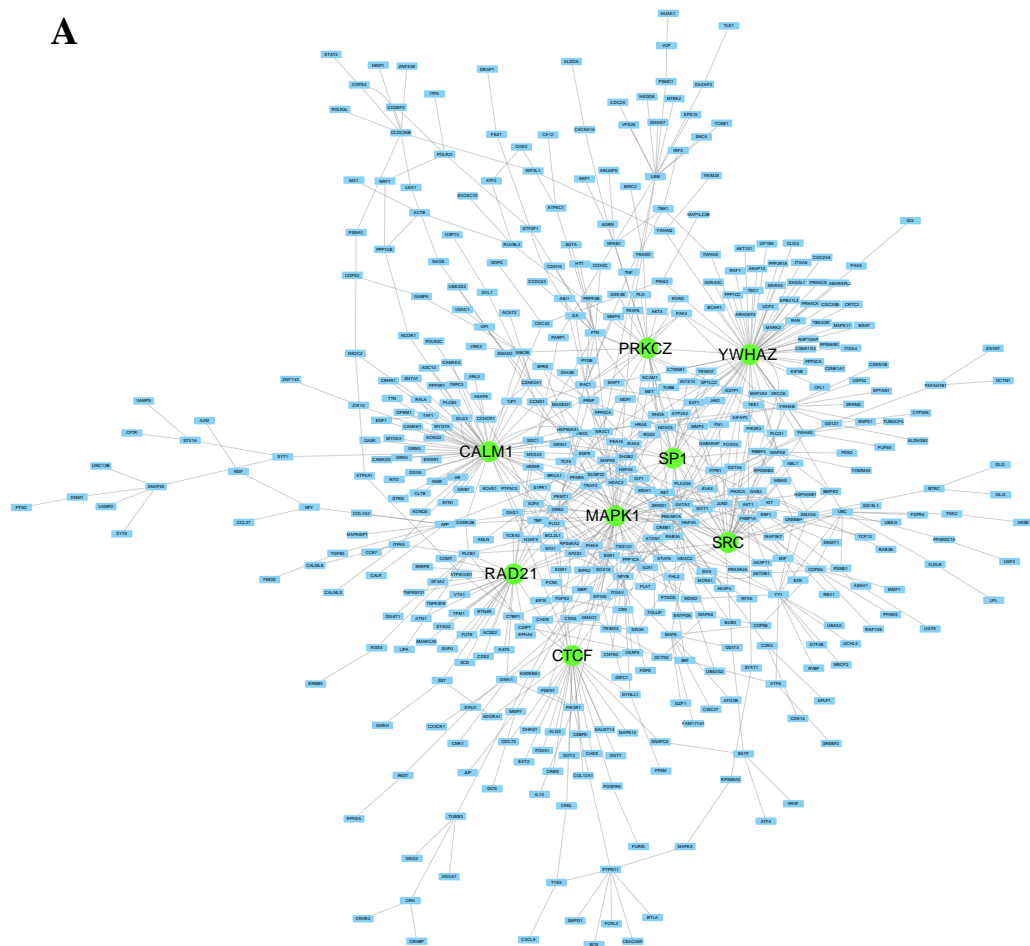

B

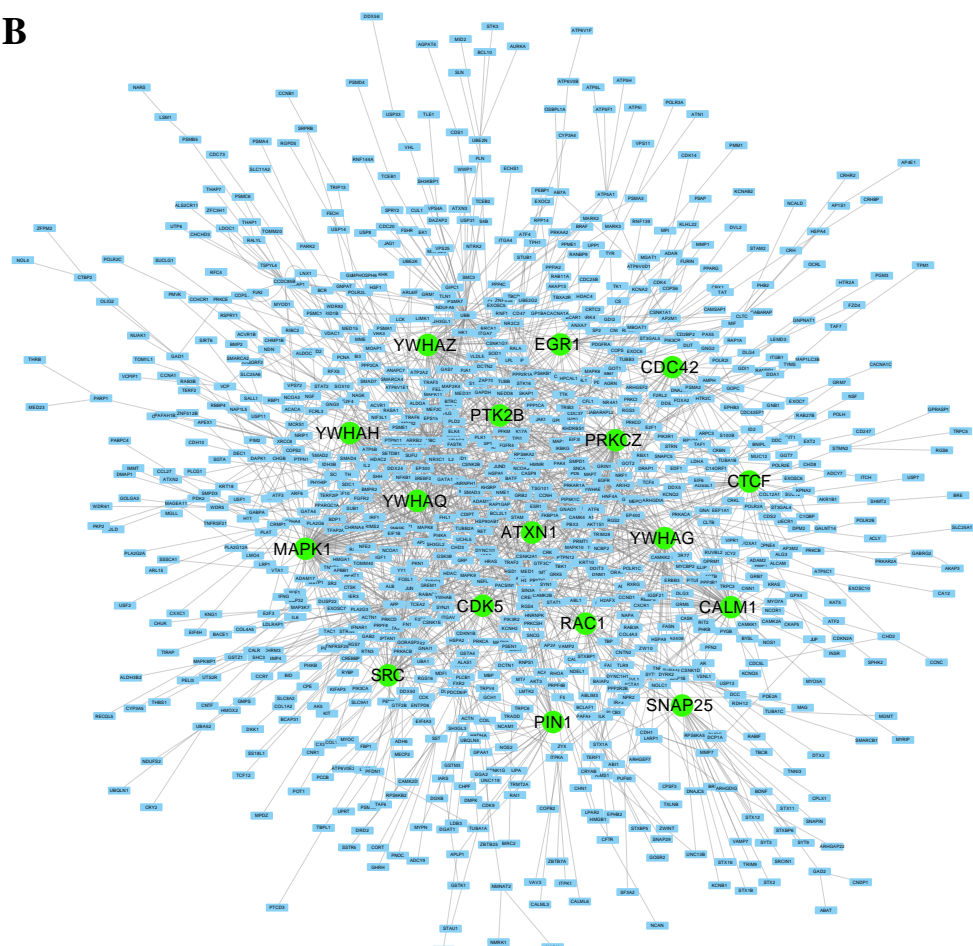

Supplement: FIGURE S6 — The downregulated (A) aging and (B) AD subnetworks. The significant interactions of young vs. aging, and young vs. AD obtained using edge betweenness network measure are shown. Genes/nodes with significant interactions are shown in green color. [file Image_6.PDF]

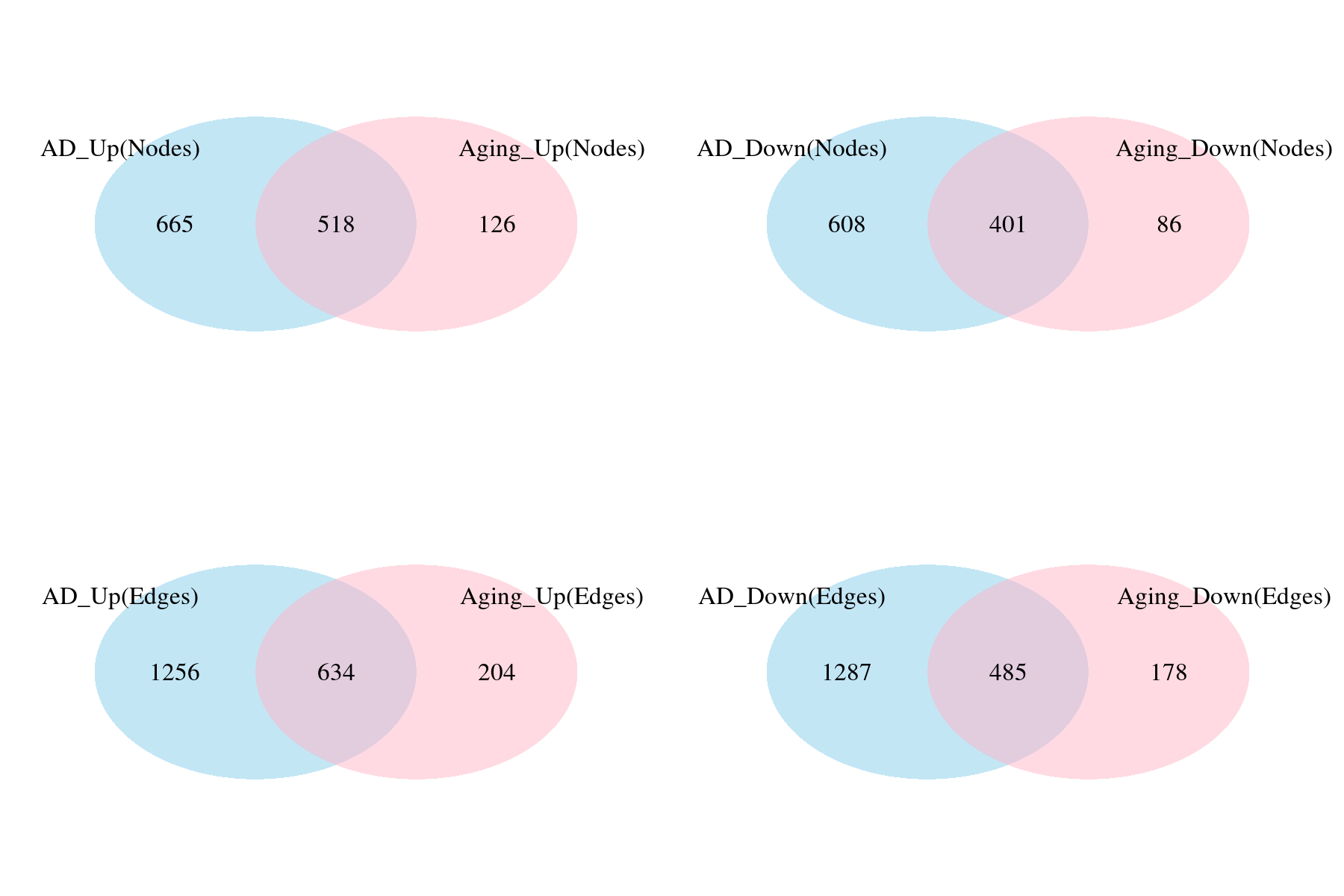

Supplement: FIGURE S7 — The overlap of upregulated and downregulated nodes and interactions between aging and AD subnetworks. [file Image_7.TIFF]
