# Supplementary material for: Integrative Analysis of Hippocampus Gene Expression Profiles Identifies Network Alterations in Aging and Alzheimer’s Disease
Source: Front Aging Neurosci. 2018 May 23;10:153. doi: 10.3389/fnagi.2018.00153 (PMC5974201; doi:10.3389/fnagi.2018.00153)
Supplement: TABLE S1 — Datasets used for the module preservation analysis. [file Table_1.DOC]

**Table S1**: Datasets used for the module preservation analysis

| **GEO Dataset** | **Platform** | **Samples (Aged+AD)** | **Normalization Function** | **R Package** |
| --- | --- | --- | --- | --- |
| GSE29378 (CA1) | Illumina HumanHT-12 V3.0 expression beadchip  (GPL6947) | 32 (16+16) | neqc | limma |
| GSE29378 (CA3) | Illumina HumanHT-12 V3.0 expression beadchip  (GPL6947) | 31 (16+15) | neqc | limma |
| GSE36980 | Affymetrix Human Gene 1.0 ST Array  (GPL6244) | 16 (7+9) | rma | oligo |
| GSE84422 | Affymetrix Human Genome U133A & U133BArray  (GPL96 & GPL97) | 29 (11+18) | rma | affy |
| GSE28146 | Affymetrix Human Genome U133 Plus 2.0 Array  (GPL570) | 30 (8 +24) | rma | affy |
| GSE5281 | Affymetrix Human Genome U133 Plus 2.0 Array  (GPL570) | 23 (13+10) | rma | affy |
