# Supplementary material for: Integrative Analysis of Hippocampus Gene Expression Profiles Identifies Network Alterations in Aging and Alzheimer’s Disease
Source: Front Aging Neurosci. 2018 May 23;10:153. doi: 10.3389/fnagi.2018.00153 (PMC5974201; doi:10.3389/fnagi.2018.00153)
Supplement: TABLE S2 — The distribution of DEGs in the different modules of co-expression network. [file Table_2.DOC]

**Table S2**: The distribution of DEGs in the different modules of co-expression network.

| **Module (genes)** | **Young-Age (569)** | **Young-AD (1980)** | **Age-AD (687)** |
| --- | --- | --- | --- |
| M1 (1595) | 9 | 25 | 10 |
| M2 (2072) | 11 | 112 | 12 |
| M3 (675) | 79 | 205 | 6 |
| M4 (701) | 190 | 203 | 0 |
| M5 (798) | 194 | 249 | 2 |
| M6 (288) | 3 | 20 | 3 |
| M7 (333) | 1 | 31 | 2 |
| M8 (695) | 2 | 51 | 81 |
| M9 (2377) | 5 | 663 | 465 |
| M10 (2660) | 6 | 229 | 77 |
| M11 (803) | 36 | 46 | 20 |
| M12 (584) | 31 | 137 | 8 |
| M13 (2496) | 0 | 1 | 0 |
| M14 (1741) | 2 | 1 | 0 |
